# Soil microbiome indicators can predict crop growth response to large-scale inoculation with arbuscular mycorrhizal fungi

**DOI:** 10.1038/s41564-023-01520-w

**Published:** 2023-11-29

**Authors:** Stefanie Lutz, Natacha Bodenhausen, Julia Hess, Alain Valzano-Held, Jan Waelchli, Gabriel Deslandes-Hérold, Klaus Schlaeppi, Marcel G. A. van der Heijden

**Affiliations:** 1 Plant–Soil Interactions, Department of Agroecology and Environment, Agroscope, Zurich, Switzerland; 2 Department of Soil Sciences, Research Institute of Organic Agriculture (FiBL), Frick, Switzerland; 3 Plant Microbe Interactions, Department of Environmental Sciences, University of Basel, Basel, Switzerland; 4 Institute of Plant Sciences, University of Bern, Bern, Switzerland; 5 Department of Plant and Microbial Biology, University of Zürich, Zurich, Switzerland; 6 Present Address: Plant Biochemistry, Institute of Molecular Plant Biology, ETH Zurich, Zurich, Switzerland

**Keywords:** Physiology, Diseases, Microbiome, Arbuscular mycorrhiza

## Abstract

Alternative solutions to mineral fertilizers and pesticides that reduce the environmental impact of agriculture are urgently needed. Arbuscular mycorrhizal fungi (AMF) can enhance plant nutrient uptake and reduce plant stress; yet, large-scale field inoculation trials with AMF are missing, and so far, results remain unpredictable. We conducted on-farm experiments in 54 fields in Switzerland and quantified the effects on maize growth. Growth response to AMF inoculation was highly variable, ranging from −12% to +40%. With few soil parameters and mainly soil microbiome indicators, we could successfully predict 86% of the variation in plant growth response to inoculation. The abundance of pathogenic fungi, rather than nutrient availability, best predicted (33%) AMF inoculation success. Our results indicate that soil microbiome indicators offer a sustainable biotechnological perspective to predict inoculation success at the beginning of the growing season. This predictability increases the profitability of microbiome engineering as a tool for sustainable agricultural management.

## Abstract (web summary)

On-farm experiments in 54 fields in Switzerland show that inoculation with arbuscular mycorrhizal fungi can promote crop yield, and inoculation success can be predicted using soil microbiome indicators.

## Main

Agricultural intensification has achieved substantial yield increases but also contributed to biodiversity loss, soil degradation, soil pollution, greenhouse gas emissions and water eutrophication^1,2^. Ensuring food security for a growing population while reducing environmental impacts poses a dual challenge to agricultural production^3^. Alternative solutions to agrochemicals are urgently needed to increase the sustainability of agriculture.

Soil ecological engineering is an important strategy to increase sustainability and reduce the need for external resources^4–6^. Promoting beneficial soil biota is an integral part of this management practice and arbuscular mycorrhizal fungi (AMF) in particular have enormous potential to play a pivotal role^4,7^. AMF belong to the phylum Glomeromycota and form symbiotic relationships with the majority of terrestrial plants. They provide the plants with nutrients in exchange for carbohydrates^8^. A range of studies have shown that AMF inoculation in the greenhouse and in the field can enhance growth of a wide range of plants, including many agricultural crops^9–11^. AMF are best known for their ability to enhance plant nutrient uptake, in particular phosphorus, but also other nutrients^8,12^. In addition, a range of studies have shown that AMF also can improve soil structure, nutrient retention in the soil^13^, reduction of greenhouse gas emission^14,15^, drought tolerance^16,17^ and disease resistance^18,19^.

Harnessing the beneficial properties of AMF can be achieved in two ways. Native AMF communities can be promoted through favourable agricultural practices such as low tillage intensity, crop diversification and organic farming^20–22^. Alternatively, AMF can also be deliberately introduced into the soil. The latter can be a valuable strategy for restoring exhausted soils with low abundance of native AMF. While positive effects are often reported in greenhouse studies, the results of field inoculations with AMF are highly variable^9,10,23^. The mycorrhizal growth response (MGR) is a metric to express the effects of AMF inoculation on crop yield^24^. Depending on the soil and plant context, MGR effects range from beneficial to detrimental^25,26^. One unsolved aspect of the variable inoculation success is that the extent to which the introduced AMF are established often remains unknown^27^. Thus, the impact of field inoculations with AMF on crop yield is highly unpredictable and their application is currently not reliable.

**Fig. 1:**
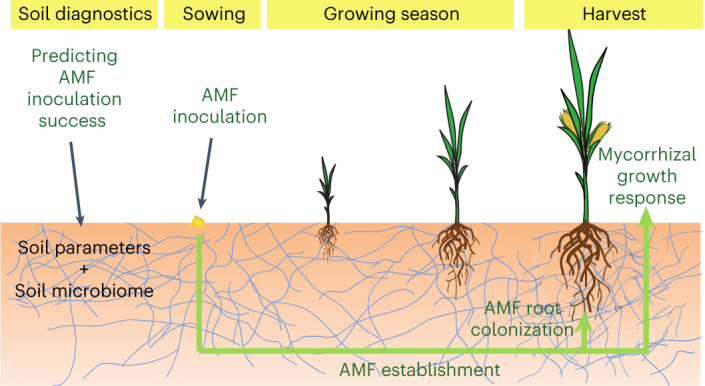
Experimental setup of the field inoculation trials. To develop a soil diagnostic tool to predict the success of AMF inoculation, a total of 52 soil parameters and the soil microbiome were analysed in 54 fields at the beginning of the growing season. After sowing, inoculations with the AMF *R. irregulare* SAF22 (or a control substrate) were carried out. At harvest, the mycorrhizal growth response, total root colonization and composition of the root microbiome were analysed to assess the success of AMF inoculation.

For AMF inoculations to become an agronomically useful management practice, reliable predictions of the conditions under which AMF enhance crop yields are urgently needed. Therefore, we conducted inoculation trials with AMF in arable fields and investigated their effect on maize growth in combination with measurements of the local chemical, physical and biological soil parameters (hereafter, ‘soil parameters’) and soil fungal microbiome, as well as changes in root microbiome composition in response to AMF inoculation (Fig. 1).

Here we aimed to test whether field inoculations with AMF are feasible and whether their success can be successfully predicted. In our proof-of-concept study, we identified the most important predictors of MGR. This will pave the way for developing a soil diagnostic tool that will inform farmers about the expected benefits of AMF inoculations in their fields, thus improving the reliability and profitability of their application.

## Results

### Maize growth responses to AMF inoculation vary strongly

**Fig. 2:**
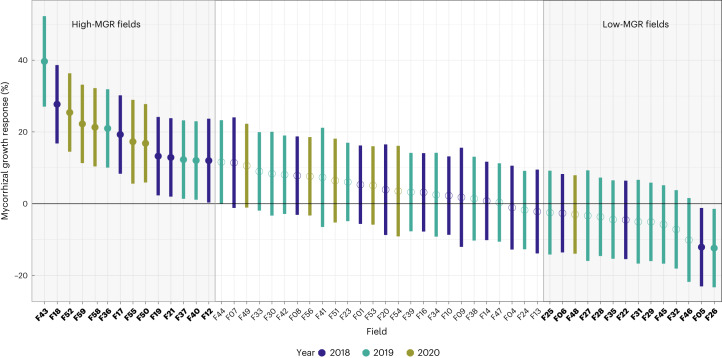
Mycorrhizal growth response over 3 yr. MGR varied widely, ranging from −12 to +40%. Plot shows mean values (circle), as well as the confidence interval of MGR for each field (*n* = 8 independent control and inoculated plots). Significant differences are highlighted by filled circles. High- and low-MGR fields (75th and 25th quantiles) are highlighted by shaded areas and bold *x-*axis labels. We observed a slight year effect. 2019 was the year with the lowest number of fields with significant positive effects (4 out of 25 fields, 16% of fields) and 2020 was the year with the highest number (5 out of 12 fields, 41% of fields). This could be due to different weather conditions during the 3 yr.

Inoculation trials with the native AM fungus *Rhizoglomus irregulare* SAF22 were carried out in 54 maize fields in Northern Switzerland and their effects on maize growth were investigated (Supplementary Table 1). The MGR varied widely, ranging from −12 to +40 % (Fig. 2). Significant positive growth responses (+12 to +40%) were observed in a quarter (14) of the fields. In two fields, a significant reduction in growth (−12 %) was observed. For downstream analyses, we categorized fields according to the 25th (<−2.4% MGR) and 75th (>12.1% MGR) quantiles of the MGR range, hereafter referred to as ‘low-MGR’ and ‘high-MGR’ fields, respectively.

**Extended Data Fig. 1:**
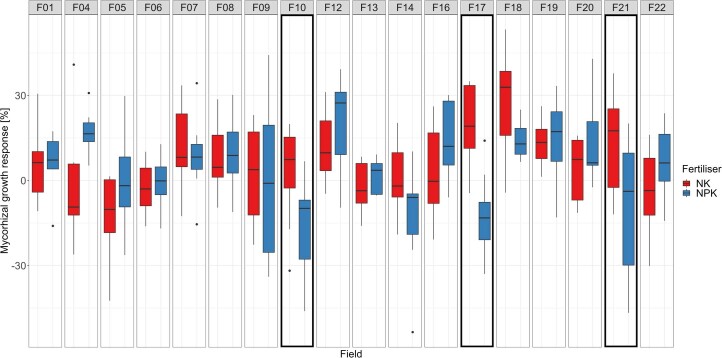
Fertilisation trial with and without phosphorus. All fields were fertilised with nitrogen (N) and potassium (K). In a subset of the fields (18 fields in 2018) we also tested whether phosphorus (P) fertilisation influenced inoculation success. Fertilisation alone had no significant effect on MGR (p = 0.3680); yet, we examined a negative interaction effect of field and fertiliser (p = 0.0001). In three fields, inoculation did not have the same effect in the presence and absence of P fertiliser, and MGR was lower in the presence of additional P (highlighted by a bold rectangle; F17: p = 0.0033, F21: p = 0.0352, F10: p = 0.0760). Fertiliser types: NK = nitrogen + potassium, NPK: nitrogen + phosphorus + potassium. The boxes represent the interquartile range (IQR, 25th to 75th percentile). The horizontal line within the box represents the median (50th percentile). The whiskers represent the minima and maxima of the data in comparison to the IQR (±1.5*IQR). Outliers are represented as individual data points.

All inoculation trials were performed without phosphorus fertilization. In a subset of the fields (18 fields in 2018), we also tested whether the addition of phosphorus influenced inoculation success (Extended Data Fig. 1). In the majority of tested fields, we did not find a significant effect of fertilizer type.

### Most important soil parameters for MGR prediction

To identify the main factors explaining the variation in MGR, we measured a total of 52 soil parameters. Field sites used in this study differed widely in terms of soil properties (for example, total phosphorus (P) content varied with a factor of 4 (from 570 to 2,312 mg kg^−1^ dry soil), mineralized nitrogen (N) content varied with a factor of 11 (from 9 to 102 mg kg^−1^ dry soil) and soil organic carbon content varied with a factor of 4 (from 0.8 to 3.4%); Supplementary Table 1). Different soil types were examined in ref. ^28^. We first assessed pairwise correlations between soil properties and MGR for 54 fields. This yielded only few and weak relationships (Supplementary Table 2).

**Fig. 3:**
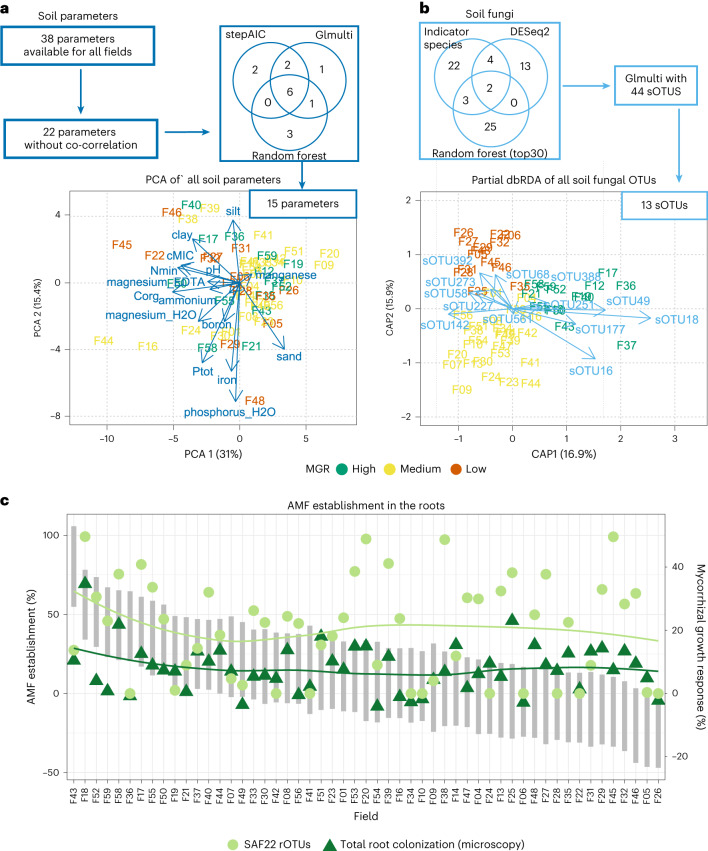
Variable selection for the MGR prediction model. **a**, Soil parameters were initially filtered through the removal of co-correlated variables, followed by random forest, stepAIC and glmulti analyses resulting in a final set of 15 variables, displayed as loading vectors in the PCA plot of all soil parameters. **b**, Soil fungal OTUs were selected using indicator species, differential abundance and random forest analyses, and a further refinement step using glmulti. This resulted in a final set of 13 sOTUs, depicted as loading vectors in the partial (adjusted for year effect) dbRDA plot, showing a clear grouping by MGR category. **c**, Establishment success of the inoculated AMF. Fields are shown in descending order of MGR (grey bars, representing the confidence interval of MGR for each field, displayed on the secondary *y* axis). AMF establishment success (displayed on the primary *y* axis) is shown as the difference (Δ) between inoculated and control samples for the relative abundances of SAF22 rOTUs and total colonization. The plot shows that there is no relationship between MGR and establishment success (indicated by the smoothed lines, which follow a different trend from that of MGR). However, the relative abundance of SAF22 and total root colonization are strongly correlated (see Extended Data Fig. 5c for pairwise correlations).

**Extended Data Fig. 2:**
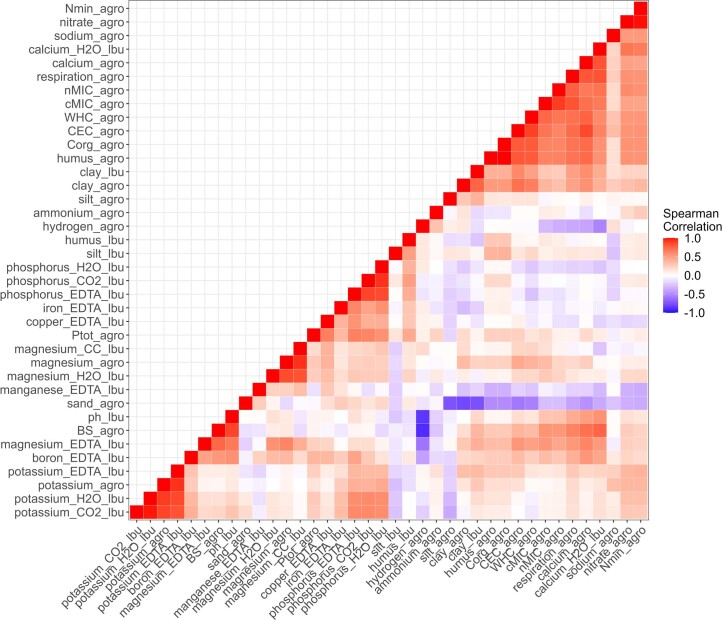
Correlation matrix of soil parameters. Matrix shows Spearman rank correlations between all 38 soil variables for which data were available for all 54 fields.

**Extended Data Fig. 3:**
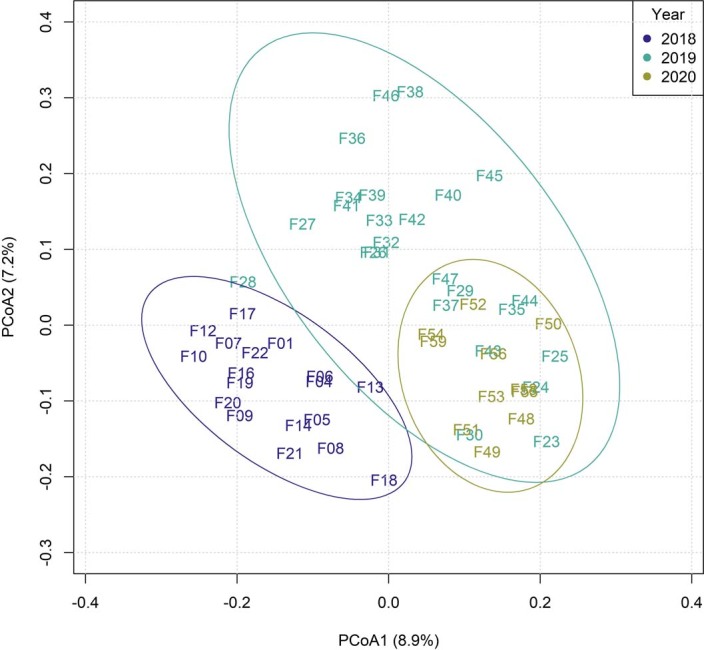
Soil fungal communities group by year. PCoA was performed on the square root transformed sOTUs based on Bray-Curtis dissimilarities. Plot shows an underlying year effect in the soil fungal data.

Because such one-factor analyses were clearly insufficient to explain MGR, we examined the relationship between soil properties and MGR with multivariate models in a second step. For this we reduced the pool of 38 parameters for which data were available for all 54 fields and filtered out strongly correlated variables (*r* > 0.8 or *r* < −0.8; Extended Data Figs. 2 and 3). This resulted in a subset of 22 variables (Supplementary Table 3). Further reductions were achieved using three different approaches including a random forest analysis^29^ (Fig. 3a and Supplementary Table 4), stepwise model selection using ‘stepAIC’^30^ and exhaustive screening using ‘glmulti’^31^ (Methods and Supplementary Table 5). The following 6 variables were identified by all approaches: magnesium (EDTA), magnesium (H_2_O), manganese, mineralized N (N_min_), iron and microbial biomass carbon (cMIC). The final pool of soil parameters for subsequent MGR prediction was derived from the combined result of all three analyses and comprised 15 variables (Fig. 3a and Supplementary Table 5).

### Most important soil OTUs for MGR prediction

We determined the soil fungal communities with long-read sequencing and found a high relative abundance of Ascomycota, followed by Mortierellomycota and Basidiomycota^28^ (Supplementary Table 6). Rarefaction analysis confirmed that sufficient sequencing depth was reached to capture the fungal diversity^28^. Unconstrained ordination revealed that soil fungal communities were grouped by year, which was subsequently included as a co-variable in all downstream analyses (Extended Data Fig. 3).

Analogous to the soil parameters, we reduced the number of fungal taxa (represented as operational taxonomic units (OTUs), hereafter referred to as sOTUs for soil OTUs) for model input using different approaches (Fig. 3b). The combined results of an indicator species analysis (Supplementary Table 7) and differential abundance analysis (Supplementary Table 8) comparing high- and low-MGR fields, as well as a random forest analysis performed on the continuous MGR values (Supplementary Table 9), yielded a total of 44 sOTUs (see Supplementary Methods for more details).

**Fig. 4:**
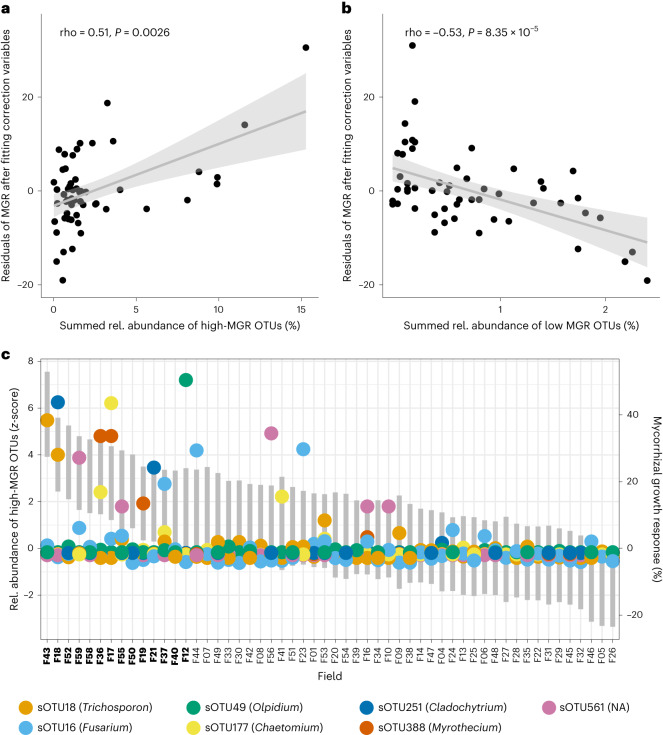
Abundance of soil fungal OTUs associated with high and low MGR. **a**,**b**, The summed relative abundances of the 7 and 6 sOTUs indicative of high (**a**) and low (**b**) MGR, respectively, correlate well with the residuals of MGR after fitting the 15 soil parameters. The correlation coefficients (Pearson, rho) and the significance values (*P*) are displayed in the plots. The regression line is shown in grey and the 95% confidence interval is the grey shaded area. **c**, The relative abundance of high-MGR sOTUs was standardized using *z* transformation for better visualization and is displayed on the primary *y* axis. The MGR range per field is indicated by grey bars (representing the confidence interval of MGR) and is displayed on the secondary *y* axis. Fields are arranged in descending order of MGR. The plot shows that on average, only one or two of these OTUs were abundant in a field with high MGR. Therefore, these predictors are only suitable in combination in a multiple linear regression model. NA, unknown. Full information on taxa identities can be found in Supplementary Table 10. The corresponding plot for low MGR OTUs can be found in Extended Data Fig. 4.

**Extended Data Fig. 4:**
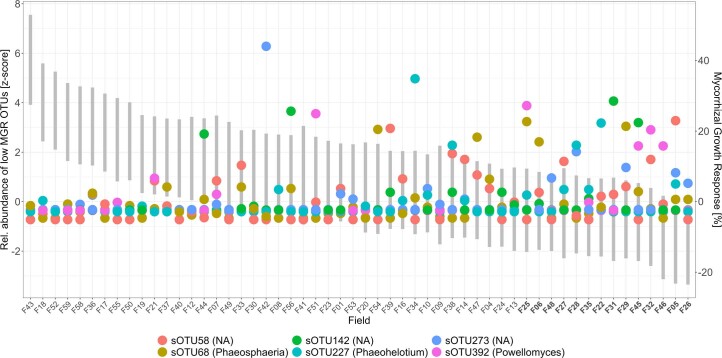
Relative abundance of sOTUs associated with low MGR across fields. The relative abundance of OTUs was standardised using z-transformation for better visualization (primary y-axis). MGR is displayed in grey bars on the secondary y-axis and fields are arranged in descending order of MGR. The plot shows that on average only one or two of these OTUs were abundant in a field with low MGR. Full information on taxa identities can be found in Supplementary Table 10.

To further refine the pool of sOTUs, another exhaustive automated model selection was performed using glmulti^31^, resulting in the selection of 7 and 6 sOTUs associated with high and low MGR, respectively (Fig. 3b and Supplementary Table 10). The genus *Phaeohelotium* was represented with two sOTUs that were associated with low MGR. In contrast, sOTUs that were more abundant in fields with high MGR included several genera with plant pathogenicity potential. These comprised *Fusarium*, *Olpidium*, *Myrothecium*, *Striaticonidium* and *Chaetomium*. The summed relative abundances of these sOTUs associated with low and high MGR each correlated well with MGR (Fig. 4a,b). In fields with high MGR, up to two indicator sOTUs for high MGR were present (Fig. 4c), while in fields with low MGR, two to three indicator sOTUs for low MGR were abundant (Extended Data Fig. 4).

### Establishment success is insufficient to explain MGR

**Extended Data Fig. 5:**
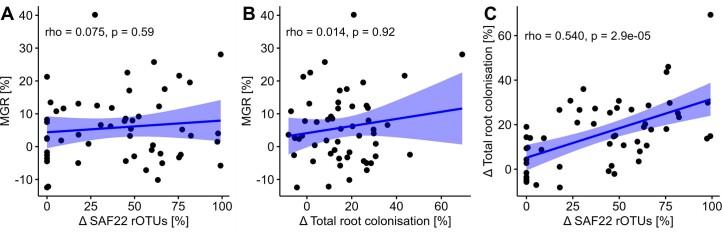
Relationship between the establishment of AMF and MGR. The relative abundance of the inoculated SAF22 was analysed using long-read sequencing. Total root colonisation was determined by microscopy. The values for the relative abundance of SAF22 and total root colonisation represent the differences between inoculated and control plants (Δ in the plots). No correlations were found between MGR and the relative abundance of sequenced SAF22 rOTUS (A) or total root colonisation (B). However, the relative abundance of SAF22 and total root colonisation were positively correlated (C). The correlation coefficients (Spearman rank, rho) and the significant values (p) are displayed in the plots. The blue shaded area represents the 95% confidence interval.

To examine the establishment of the AMF inoculum in the maize roots, we measured total root colonization by microscopy and establishment success of the inoculated strain SAF22 by profiling the fungal root microbiome using long-read sequencing (root (r)OTUs). However, none of these parameters correlated with MGR (Fig. 4c and Extended Data Fig. 5).

**Extended Data Fig. 6:**
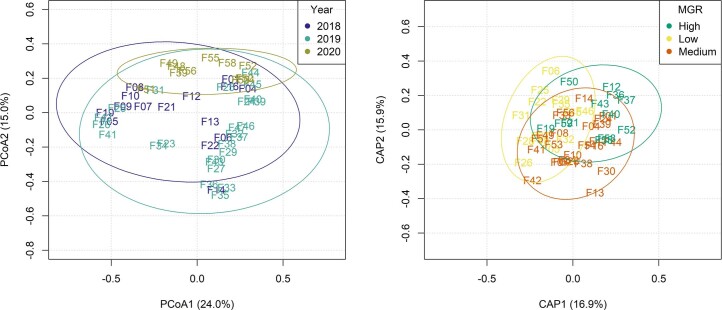
Ordination plots of native root fungal communities. In order to explore a possible year effect and the relationship between the overall community composition and MGR, ordinations were performed on the control samples. The principal coordinate analysis on the left shows only weak grouping by year. Similarly, the partial distance-based redundancy analysis, after partialling out the effect of the variable year, only shows weak grouping by the response variable MGR. Ordinations were performed on the square root transformed rOTUs based on Bray-Curtis dissimilarities.

**Extended Data Fig. 7:**
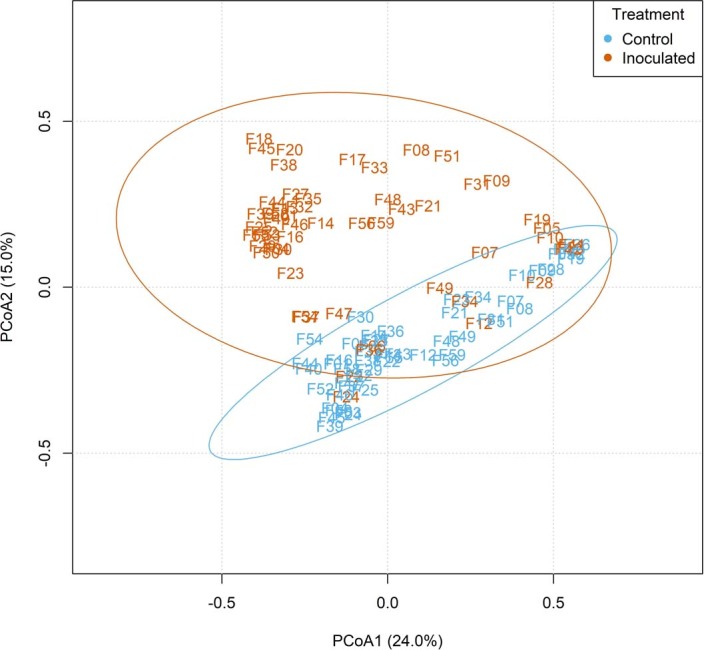
Ordination of control and inoculated root microbiome. The principal coordinate analysis shows clear separation between the control and inoculated samples, indicating that the inoculated SAF22 established well in most fields (see Supplementary Table 12 for relative abundance values of each field), and thus, causing a shift in the root microbiome. Ordination was performed on the square root transformed OTUs based on Bray-Curtis dissimilarities.

The fungal root microbiome was determined using PCR primers that enrich for AMF (Supplementary Fig. 1 and Table 11). Rarefaction analysis confirmed that sufficient sequencing depth was reached (Supplementary Fig. 2). Similar to the soil microbiome, albeit less pronounced, the native root microbiome (control samples) showed a year effect (Extended Data Fig. 6). With the ratio between inoculated and control samples, we quantified the establishment success of the inoculum *R. irregulare* SAF22, which ranged between 17.8% and 100% in 38 fields (Supplementary Table 13). In 5 fields, establishment was very low (0.7–9.4%), while in 11 fields, it did not establish at all (0%). Overall, we found clear differences between control and inoculated samples (Extended Data Fig. 7 and Supplementary Fig. 3).

### Soil pathogenic fungi are most important predictors of MGR

**Fig. 5:**
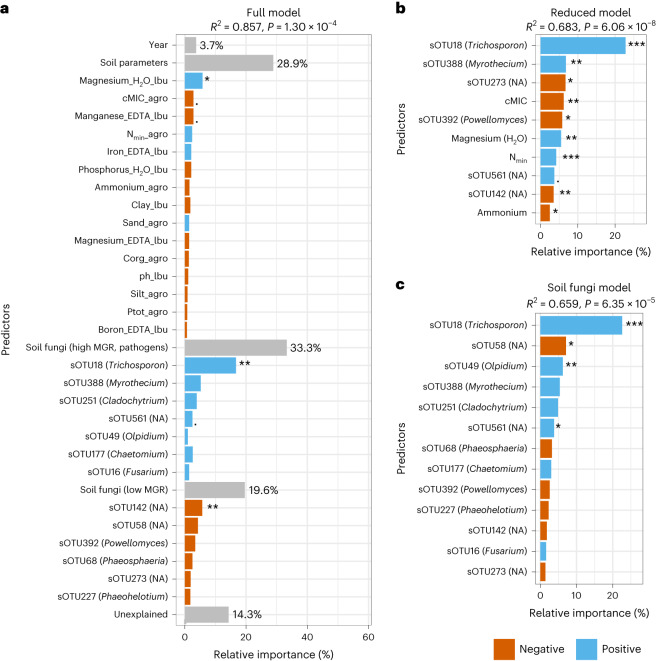
Relative importance of the most important predictors of MGR. Predictors are displayed on the *y* axis. The combined relative importance of soil parameters and sOTUs associated with low and high MGR is summarized in grey bars. A higher value of a predictor in blue is associated with a higher MGR, while a higher value of a predictor in red is associated with a lower MGR. The genus identity of the OTUs is given in brackets. **a**, Full model with 15 soil parameters, 13 soil fungal OTUs and year. The growth responses of maize to mycorrhizal inoculation were best predicted by the soil fungi that were present in the fields (53%) and to a lower degree by the soil parameters (29%). **b**, Reduced model with the top 10 predictors. **c**, Soil fungal model. *F*-test: ***P < 0.001, **P = 0.001–0.01, *P = 0.01–0.05, ‘.’*P* = 0.05–0.1.

For prediction of MGR, we then modelled all the previously selected 15 soil parameters and 13 sOTUs (Fig. 5). Combining these predictors in a full model, they were able to explain 86% of the variation in MGR (*P* < 0.001; Fig. 5a). Interestingly, the 15 soil parameters were by far less important (29%) than the 13 sOTUs (53%). A reduction of predictors, comparing all possible models with a maximum size of 10 predictors, resulted in a model consisting of 3 high-MGR sOTUs (*Trichosporon*, *Myrothecium*, unknown), 3 low-MGR sOTUs (*Powellomyces*, two unknown), as well as N_min_, cMIC, ammonium and magnesium (H_2_O) (Fig. 5b). Although highly simplified, this model was still able to explain 68% of the variation in MGR (*P* < 0.001). An alternative model with only the 13 sOTUs (Fig. 5c) was almost as good, reaching 66% explanation (*P* < 0.001).

**Extended Data Fig. 8:**
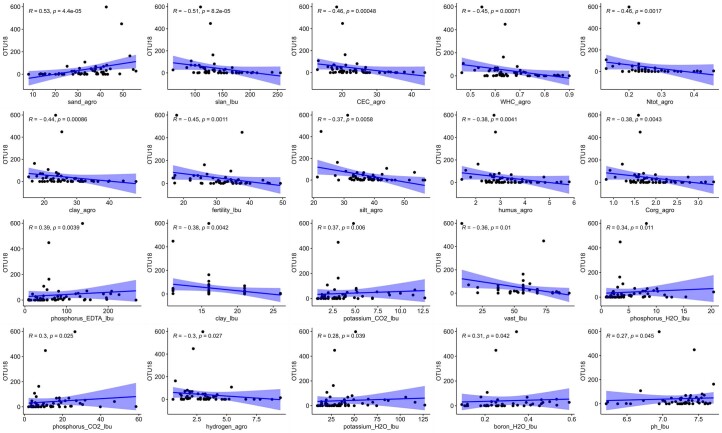
Significant correlations of soil parameters with the most important sOTU associated with high MGR. sOTU18 (Trichosporon sp.) showed the highest relative importance in the MGR prediction model. Its abundance correlates strongly with properties of poor soils (that is, lower organic carbon and nutrient contents and higher sand fraction). The correlation coefficients (Spearman rank) and the significant values (p) are displayed in the plots The blue shaded area represents the 95% confidence interval.

sOTU18, identified as *Trichosporon* sp., was the most important predictor for high MGR in all models. Species of the genus *Trichosporon* are known pathogens; however, there are currently no reports on plant pathogenicity. Interestingly, the abundance of sOTU18 correlated with variables indicative of low-carbon and low-nutrient fields (Extended Data Fig. 8).

We followed a binary classification approach to cross-validate our models since the ultimate goal is not to predict the exact value of the MGR, but to provide recommendations on whether inoculation provides a benefit (significant positive growth response, >12.2%, as this was the lower limit for significant positive effects in this study) or not (neutral or negative growth response, <12.2%). This resulted in a high mean accuracy of 80% (full model) and 83% (reduced model and soil fungus model). We are thus able to make the right decision (to inoculate or not) with a high probability.

### Root microbiome data confirm results of prediction model

**Fig. 6:**
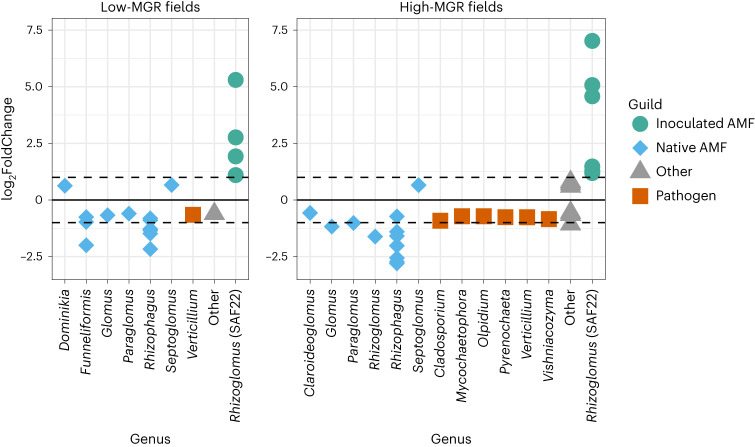
Comparison of differentially abundant rOTUs between control and inoculated samples for fields with low and high MGR. Differential abundances were assessed using DESeq2 (Wald test; significance threshold, 0.1; *P* values adjusted for multiple comparison; dashed lines correspond to a log_2_FC of −1 and 1 to guide the eye). In fields with low MGR (left), the inoculated *R. irregulare* SAF22 (represented by several OTUs corresponding to rRNA variants, see Methods) replaced the native AMF, while in fields with high MGR (right), not only the native AMF but also pathogenic fungi were replaced. Full taxonomic assignments of the OTUs can be found in Supplementary Tables 14 and 15. It should be noted that primers targeting AMF were used for the root data, so a number of fungi could not be detected, including some pathogens (for example, *Fusarium*, *Myrothecium*) that had been identified as significant in the soil data. Of note, we identified *Trichosporon*, the most important soil fungal OTU in the predictive model, also in the data of the root fungal communities (rOTU20 shares 100% sequence similarity with sOTU18 over its entire amplicon length). Even though it does not appear in the plot of fields with high MGR as it was below the significant threshold (*P* = 0.252), it showed a similar trend (log_2_FoldChange = −0.583) as the displayed pathogens (Supplementary Table 15).

To investigate the relationships between plant pathogenic soil fungi and the inoculated AMF, we investigated the root fungal profiles for community shifts in response to inoculation. We performed a differential abundance analysis comparing control vs inoculated plots in low-MGR fields and control vs inoculated plots in high-MGR fields. In low- as well as high-MGR fields, we find a lower relative abundance of several native AMF (including the genera *Funneliformis*, *Rhizophagus*, *Glomus* and *Paraglomus*; Fig. 6, and Supplementary Tables 14 and 15) in the plots inoculated with *R. irregulare* SAF22. It is important to note that AMF inoculation changed community composition, but it had no significant effect on AMF diversity (AMF OTU richness increased in 42% of the fields and decreased in 44% of the fields upon inoculation; Supplementary Table 16).

Even more remarkably, in fields with high MGR, the introduced strain SAF22 also reduced the relative abundance of several plant pathogenic taxa. These included the genera *Olpidium*, *Cladosporium*, *Mycochaetophora*, *Pyrenochaeta* and *Vishniacozyma* (Fig. 6 and Supplementary Table 15). Taken together, the in-depth analysis of the root fungal profiles revealed a plausible mechanistic basis for positive MGR: the introduced AMF outcompetes the otherwise plant pathogenic fungi from the roots most probably resulting in better growth of the maize plants.

## Discussion

Here we show that inoculation with arbuscular mycorrhizal fungi significantly increased maize yield. We achieved a significant positive increase in biomass of 12–40% in a quarter of the fields, which is considerably higher than the annual yield increases through breeding for a range of crops (which are often below 1%)^32^. Moreover, effect sizes of adding cover crops (up to 8%)^33^ and other biofertilizers (up to 12%)^34^ in comparable climatic regions and production systems are also lower compared with growth increases in inoculated high-MGR fields in this study.

While many studies pointed to the importance of AMF for plant nutrition, this study links AMF inoculation to soil pathogen protection. Pathogen abundance in the soil best explained AMF inoculation success (33% of variance explained), while soil parameters were less important (29%; Fig. 5a). While a range of studies have shown that inoculation with AMF can promote plant growth in the field^9–11^, results are variable and none have used soil characteristics and molecular-based soil microbiome analysis to specifically predict under which conditions AMF can promote plant growth.

Phosphorus availability tended to be negatively associated with inoculation success in previous studies^35^. In our study, phosphorus explained less than 2% of the variation in MGR, which was also reflected in the outcome of the fertilizer trial (see Supplementary Results and Extended Data Fig. 1). Despite a large (factor of 26) variation in immediately plant-available phosphorus levels (0.34–9.07 mg kg^−1^, H_2_O-CO_2_ extraction; Supplementary Table 1), most soils were above the threshold for phosphorus deficiency in Swiss soils (0.58 mg kg^−1^)^36^, perhaps also explaining why AMF inoculation success was best explained by other factors.

Further, positive growth responses were associated with lower soil organic carbon levels and especially with reduced soil microbial biomass carbon (Fig. 5b). Soil microbial biomass represents the living fraction of organic carbon and is an important component of soil health^37,38^. Fields with low microbial carbon content appeared to benefit more from AMF inoculations, suggesting that AMF are particularly important when soil health is low. It is also known that organic amendments can suppress a wide range of pathogens in the soil^39,40^. Therefore, protection from pathogens by inoculated AMF may be particularly important in soils with low organic content. Consequently, AMF inoculations in healthier soils with high abundance of OTUs associated with low MGR (for example, *Phaeohelotium*; Supplementary Table 10) are less likely to provide economic benefits.

Several sOTUs associated with high MGR in this study are known as plant pathogenic taxa (Supplementary Table 10) and can infect important crops including maize^41–45^. These comprise *Olpidium brassicae*^41,42^, *Myrothecium* sp.^43^ and *Fusarium equiseti*^44,45^. The most important predictor in the model, however, was sOTU18 with the genus assignment *Trichosporon*, known to cause diseases in human^46^. So far, this genus has not been described in relation to plant pathogenicity; yet, it best explained inoculation success with AMF and especially in high-MGR fields where it was less abundant in inoculated plots, AMF had a positive impact on plant yield (Supplementary Tables 14 and 15), suggesting a negative effect of this taxon on maize growth. Moreover, sOTU18 is an indicator of poorer soil properties (that is, negatively correlated with organic carbon and soil fertility, and positively correlated with sand content; Extended Data Fig. 8). Overall, pathogen abundance might be more pronounced in poorer soil. The addition of AMF provides additional protection and plants growing in these fields might benefit more from mycorrhizal inoculation. Given the limitations of marker genes in predicting fungal lifestyles, further studies need to isolate these fungi and test whether they indeed negatively affect maize growth to experimentally verify their pathogenicity potential and to what extent AMF can contribute to pathobiome management.

Only few pathogens seem to be important in the studied context, as the summed abundances of all soil fungal pathogens identified by guild-based screening was not able to predict MGR (see Supplementary Results and Extended Data Fig. 4). AMF strains are probably specialized in their ability to protect against specific pathogens. Here we inoculated an AMF strain that was isolated from Swiss soil and can establish well in a wide range of soil types^24,35^. The inclusion of other AMF genotypes to be screened for their properties to protect against specific pathogens would not only broaden the scope of this management practice and facilitate establishment under a wide range of conditions, but could also prevent possible agricultural intensification and biodiversity loss through the employment of only one AMF strain. Even though we did not observe a reduction in AMF diversity (Supplementary Table 16), future studies need to investigate the long-term effects of inoculations, as well as the persistence and invasiveness of native vs exotic AMF inocula. The unintended consequences of non-native inoculants in natural and agricultural systems are not known, but if inoculants are invasive, they may pose a threat to soil and plant biodiversity and ecosystem functioning^47^. Furthermore, complementary to more diverse and complex inocula, the possibility of AMF rotations—analogous to crop rotations—could also mitigate the risk of low-diversity microbial treatments on soil biodiversity.

The ability of AMF to protect plant roots from attack by soil-borne pathogens can be explained by various mechanisms including improved plant nutrient uptake and consequently plant health^48^, induced systemic resistance^49^, alteration of the root microbiome^50^ and direct competition for root space^18,19,51^. In our study, several of these mechanisms of action probably occurred simultaneously. Our root microbiome data partly point to direct competition for root colonization. In fields with high MGR, pathogenic fungi were significantly less abundant in inoculated roots (Fig. 6). These included the previously identified important soil pathogens *Olpidium* and (potentially plant pathogenic) *Trichosporon*, as well as *Cladosporium*, *Mycochaetophora*, *Pyrenochaeta* and *Vishniacozyma*. *Myrothecium* and *Fusarium*, which were also identified as important predictors, could not be found in the root microbiome data, possibly because the molecular primers used for roots were specifically designed to target AMF^52^. Thus, general ITS (internal transcribed spacer) primers for the roots also need to be included in future studies to cover full fungal diversity.

Moreover, it was striking that there was no correlation between root colonization and plant growth response. Inoculation of the AMF strain SAF22 was the experimental factor, but inoculum success and how well the AMF strain established was not a good predictor. Instead, differences in its functions explained the variation in MGR. In contrast to the common interpretation where biofertilizers stimulate plant growth, here the interpretation is the other way around: abundant pathogenic soil fungi, which are present in ‘high-MGR’ fields, cause a growth reduction in the control treatment, while this otherwise negative effect is mitigated by the inoculated AMF. We believe this could be due to several reasons closely related to the many ways AMF can suppress pathogens. First, if AMF establish first and fast, this could prevent or reduce pathogen establishment. Second, a range of studies have shown that AMF can trigger induced systemic resistance^49,53–55^, and AMF may indirectly affect pathogens by altering the microbiome^50^. Time-resolved studies that follow the processes and mechanisms in the roots throughout the growing season are needed.

Several studies have shown that the abundance and activity of AMF are also explained by the bacterial microbiome^56^ and pesticide application^57,58^. Further, differences in microclimatic conditions may be another factor contributing to differences in MGR. The inclusion of such factors may resolve even more of the unexplained variance. However, while field inoculations must be economically viable, simple and cost-effective prediction of inoculation success must also be possible. Predicting MGR based solely on sequencing soil fungal pathogens, for instance, would represent a simplified diagnostic approach. Here we present an initial list of pathogenic sOTUs that could be quantified directly in the field at the beginning of the growth season, with results being available within a few hours and at a reasonable cost using quantitative PCR or rapid sequencing^59^. Furthermore, automated and affordable microbial diagnostic assays could be developed (for example, Loop-mediated isothermal amplification). Subsequently, pathogen abundance can predict inoculation success.

With this work using 54 fields, we have shown that field inoculation with AMF can successfully be predicted and can give a yes/no recommendation with high accuracy of 80–83%; this means a successful prediction in 4 out of 5 fields. We have solved the context dependency for one maize variety in one geographic area. The approach presented here is easily transferable and further studies need to test different maize varieties, as their responsiveness to mycorrhiza can vary greatly^23,60^. The inclusion of a broad range of soil types and climatic zones will further extend the scope of the work. To maximize the potential of AMF for more sustainable agricultural production systems, future work needs to include settings with reduced use of agrochemicals. Furthermore, our approach can be used as a blueprint to predict inoculation success and resolve context dependency of other widely used biofertilizers including *Rhizobium* spp. or *Bacillus amyloliquefaciens*^61^.

With our results, we provide a crucial starting point for the development of a diagnostic tool using soil microbial indicators that can ultimately increase the reliability of field inoculations. As a result, AMF inoculations can become a powerful management option for microbiome engineering in arable land and thus an integral part of agricultural sustainability.

## Methods

### Field sites

The field inoculations were carried out in three consecutive years in a total of 54 maize fields in northern Switzerland. In 2018, 22 fields were inoculated between 23 April and 16 May. In 2019, 25 fields were sampled between 18 April and 7 June. In 2020, 12 fields were sampled between 22 April and 16 May. The exact GPS locations are available but are not provided here for confidentiality reasons. The farms were chosen on the basis of the farmers’ willingness to participate in this study and planned cultivation of maize for the respective growing season. Apart from inoculum and fertilizer application, the experimental sites were managed by the farmers according to Swiss standards of conventional farming^62^.

### Fertilizer

All fields were fertilized with N and potassium (K). Since it has previously been shown that MGR is negatively correlated with the fertilized amount of P^35^, a subset of the 2018 plots (see below) were additionally fertilized with P to further verify these results. The amount of N, K and P was calculated on the basis of the Principles of Agricultural Crop Fertilisation (PRIF) in Switzerland^62^, which gives recommendations on the amount of fertilizer to be applied based on the plant and its specific nutrient needs. The following granular fertilizers were used: N in the form of 24% ammonium nitrate (NH_4_NO_3_), P as triple superphosphate (46% P_2_O_5_) and K as 60% water-soluble potassium oxide (K_2_O). The correct amount of fertilizer that was going to be applied (20.1 g N, 8.2 g of P_2_O_5_ and 22.1 g K_2_O per m^2^) was filled into sealed bags.

### Experimental setup

In 2018, a split plot design was used for practicality reasons. Each experimental field comprised 12 maize rows of 24 m length, with the spacing between two maize rows being 75 cm. Fertilizer types (NK and NPK) were randomly assigned to whole plots, and inoculum types (control and AMF) were randomly assigned to split plots within each whole plot. A total of 16 whole plots was installed in a square 2 × 8 design, each of them comprising an area of 13.5 m^2^ (corresponding to 6 maize rows of 3 m length). Each whole plot contained an AMF and a control treatment, separated by two maize rows, resulting in eight replicates per treatment combination. In 2019 and 2020 a randomized complete block design was used with 8 blocks. It was ensured that there were at least three maize rows serving as a buffer zone between the first inoculated maize row and the edge of the field, to avoid edge effects.

### Inoculation

The control (carrier substrate) and AMF inocula (*R. irregulare* isolate SAF22) were produced in the greenhouse. *Plantago lanceolata* L. was planted in 7 l pots filled with an autoclaved soil:sand mixture (3:17 v/v)^35^ and inoculated with SAF22 or no AMF (control). The pots were watered regularly and after 3 months, the watering was stopped and the pots dried out. The resulting mixture of sand, soil, roots and AM fungal spores was used to inoculate the fields.

The maize variety LG 30.222 (UFA) was chosen on the basis of its high responsiveness to SAF22 in a previous study^35^. In addition, in 2019, an inoculation trial with individual and combined inoculations of different AMF species (*R. irregulare* SAF22, *Funneliformis mosseae*, *Clareoideoglomus claroideum*) was carried out on a subset of 10 fields (Extended Data Fig. 10).

After sowing, inoculations were performed as soon as possible (after 2–7 d), except for fields F10, F16 and F17 (9–11 d) due to late notice by the farmer. In each plot, the farmers’ seeds were carefully removed and the seed furrow was dug out to ~15 cm deth and 15 cm width. The soil and the respective inoculum (control or AMF) were alternately filled back into the hole and mixed well. A stretch of 80 cm in a maize row was inoculated with 450 g of the respective inoculum, which corresponds to an inoculum concentration of ~5% (v/v). Seeds of the maize variety LG 30.222 (UFA) were placed back into the soil–inoculum mix in their former position and covered with soil. Within the inoculated stretch, five seeds were placed ~3–4 cm deep in the soil with a 15 cm spacing between them and loosely covered with soil. The seeds were coated with standard fungicides as well as the insecticide and bird repellent Mesurol, as this is common practice in conventional farming in Switzerland. We controlled for possible adverse effects of the fungicide coating on the AMF by using the same coating in all fields. To avoid contamination, all equipment was used only for either control or AMF inoculations, and control plots were set up before AMF plots.

### Soil sampling and processing

Soil sampling took place before fertilization of the fields and was performed using a half-cylindrical gouge auger (Eijkelkamp; effective auger body 100 cm, Ø 3 cm). Twenty soil cores were mixed to form composite samples and kept cold during transportation back to the laboratory. Samples were stored at 4 °C for a maximum of 2 weeks before sieving to 2 mm. A subsample was stored frozen for DNA extraction.

A total of 52 soil analyses were carried out in the Environmental Analytics lab and Soil Biology lab at Agroscope as well as the LBU (laboratory for soil and environmental analytics) according to their standard protocols. All data are provided in Supplementary Table 1.

### Harvest

Shortly before the farmer’s planned harvest, after ~4–5 months of plant growth, two plants from the centre of each of the eight plots per treatment and field (that is, 16 plants in total) were cut 10 cm above the soil surface and their fresh weight was determined. The plants were dried at 60 °C until they reached a constant weight. Dry plant biomass was used for the predictions as maize is mostly grown for silage in Switzerland. Both parameters are strongly correlated (rho = 0.73; Supplementary Fig. 5).

Roots were collected and thoroughly washed with water, cut into pieces of ~1–2 cm length and mixed well. Subsamples of the roots for DNA extraction were stored at −20 °C. For assessment of total root colonization by microscopy, roots were stored in 50% ethanol until staining.

### General statistics and graphics

All statistical analyses described below were carried out in R (v.4.0.3)^63^ and plots were created using the R packages ggplot2 (v.3.3.5)^64^, graphics (v.4.0.3)^63^ or ggpubr (v.0.4.0)^65^. Inkscape (v.092)^66^ was used to finalize Fig. 3.

### MGR

MGR was calculated as previously described^24^ to evaluate the percentage change in maize biomass in AMF-inoculated plots in relation to the average biomass in control plots. The 25th and 75th quantiles of the MGR range were calculated and fields were grouped by MGR categories comprising low- (bottom 25%), medium- (intermediate 25%–75%) and high- (top 25%) MGR fields (Fig. 1 and Supplementary Table 1).

Differences in MGR between the subset of fields fertilized with and without phosphorus were assessed using a two-way analysis of variance (ANOVA) using the ANOVA function of the R package stats (v.4.0.3)^63^ with the two grouping variables ‘field’ and ‘fertilizer’ and their interaction effect (Extended Data Fig. 1).

### Analysis of soil predictors

To find pairwise correlations among all 52 measured soil parameters and MGR, Spearman rank correlations (corr.test function of the R package psych (v.2.1.9))^67^ were calculated and corrected for multiple testing using the Benjamini–Hochberg method^68^ (Supplementary Table 2).

Further, we performed multiple linear regression analysis. To assess the most important predictors for MGR, a stepwise reduction of parameters had to be performed. Only parameters that were measured in all 54 fields were selected (Supplementary Table 1). First, strongly correlated parameters (*R* < −0.8, *R* > 0.8) were assessed using the cor function in the R package stats (v.4.0.3)^63^, visualized in a heat map using the R packages reshape2 (v.1.4.4)^69^ and ggplot2 (v.3.3.5)^65^ (Extended Data Fig. 2), and subsequently reduced (Supplementary Table 3).

Further analyses were conducted to identify the most important predictors of MGR from this pool. Random forest analysis was performed using the R package randomForest (v.4.6-14)^29^ and 1,000 trees. The importance of predictors was ranked on the basis of their IncNodePurity values (Supplementary Table 4). Stepwise model selection was performed using the function stepAIC of the R package MASS (v.7.3.54)^30^, including backward and forward selection. The selected predictors can be found in Supplementary Table 5. To compare all possible models and identify the best model from this pool, the R package glmulti (v.1.0.8)^31^ was used with exhaustive screening, the Akaike information criterion with correction for small sample sizes (aicc) and a maximum model size of 10 predictors (Supplementary Table 5). The combined output of these analyses was used as input for the final model selection (see below).

Principal component analysis was performed using the prcomp function in the R package stats (v.4.0.3)^63^. A biplot was created with the loading vectors corresponding to the 15 selected parameters of the glmulti, stepAIC and randomForest analyses described above (Fig. 3a).

### Soil microbiome sequencing

Samples for DNA extraction were stored at −20 °C. Details of DNA extraction, PCR, library preparation and sequencing have been previously described^28^. DNA was extracted from four subsamples from each field using the NucleoSpin soil kit (Macherey-Nagel) and ~250 mg of soil. The entire ITS region was amplified using primers ITS1F (5′-CTTGGTCATTTAGAGGAAGTAA-3′)^70^ and ITS4 (5′-TCCTCCGCTTATTGATATGC-3′)^71^, employing a two-step PCR protocol. First, the ITS region was amplified from genomic DNA. A 5 Prime Hot Master Mix (Quantabio) with a total reaction volume of 20 μl containing 0.3% BSA and 500 nM of each primer was used. The PCR programme consisted of an initial denaturation step of 2 min at 94 °C, followed by 25 cycles of denaturation at 94 °C for 45 s, annealing at 55 °C for 1 min and elongation at 72 °C for 1 min, with a final elongation step of 10 min at 72 °C. Cleanup was followed by reversible solid-phase immobilization with SPRIselect beads (Beckman Coulter). In the second PCR step, barcoded ITS1F and ITS4 primers were used but without the addition of BSA. The same PCR programme was used, but with only 10 steps. DNA was quantified using Picogreen and pooled in equimolar ratios. Four libraries were prepared in the same way and sequenced using the PacBio single molecule real-time (SMRT) technology^72^. The raw data were converted to circular consensus sequences (min. passes = 5) and demultiplexed with SMRT software (v.9.0.0, Pacific Biosciences). The raw sequencing data are stored in the European Nucleotide Archive (http://www.ebi.ac.uk/ena) under accession number PRJEB53587.

### Root microbiome sequencing

The DNA from roots from each block was extracted separately according to ref. ^72^ with some modifications. After lyophilisation, roots were ground with glass beads (1 mm and 0.1 mm) in a tissue lyser (FastPrep-24) twice for 1 min at 6 m s^−1^. The NucleoSpin soil kit (Macherey-Nagel) was used as before to extract DNA from the powdered roots (~50 mg) using buffer SL1. After quantification with AccuClear Ultra High Sensitivity dsDNA quantification kit (Biotium), DNA was diluted to 1 ng µl^−1^. Samples from all the blocks for one treatment of one field were pooled using equal amounts.

We used primers that target AMF so that the community profiles included Glomeromycota besides Ascomycota and Basidiomycota taxa. A ~1.5-kb fragment was amplified using the wobble-containing variants^52^ of the AMF-specific primers SSUmCf (5′-TATYGYTCTTNAACGAGGAATC-3′) and LSUmBr (5′-AACACTCGCAYAYATGYTAGA-3′), spanning part of the small ribosomal subunit, the entire ITS region and part of the large ribosomal subunit^73^. In contrast to the original paper, the PCR method was improved by adding Q-Solution (Qiagen) and by using touchdown PCR. The polymerase system was Phusion High-Fidelity DNA system (Thermo Scientific). Two-step PCR was used to prepare the library for sequencing: the first step amplified the target gene, the second PCR used primers with a barcode specific for each sample. Reactions of the first step were prepared in 20-µl volume with HF Phusion buffer, 500 nM of each primer, Q-Solution diluted 4 times and 2 ng of DNA. Cycling programme of the first PCR consisted of an initial denaturation at 98 °C for 3 min, 10 touchdown cycles (30 s at 98 °C, 45 s annealing with temperature starting from 65 °C and reducing to 55 °C with 1 °C less per cycle, 1 min at 72 °C), followed by 25 cycles of standard PCR (10 s at 98 °C, 30 s at 55 °C, 1 min at 72 °C) and a final elongation of 10 min at 72 °C. PCR products were cleaned up using solid-phase reversible immobilization SPRIselect beads (Beckman Coulter). Reactions for the second step were prepared in 30-µl volume with the same concentrations of barcoded primers but without Q-Solution and 5 µl of the PCR product from the first reaction. The cycling programme of the second step consisted of an initial denaturation of 2 min at 98 °C, 10 cycles (10 s at 98 °C, 30 s at 55 °C, 1 min at 72 °C), followed by 10 min final elongation. After final cleanup with SPRIselect beads, DNA was quantified with AccuClear Ultra High Sensitivity dsDNA quantification kit and pooled in equimolar fashion.

Negative controls did not result in any amplification. A positive control of the inoculated AMF was included to identify OTUs corresponding to *R. irregulare* SAF22 and identified 4 abundant and 3 rare variants of the ribosomal (r)RNA operon. The resulting amplicons were sequenced in three libraries using the PacBio SMRT technology. The circular consensus sequences are stored in the European Nucleotide Archive (http://www.ebi.ac.uk/ena) under accession number PRJEB56590.

### Bioinformatics

Samples for each library were analysed together to form one OTU table. Samples were demultiplexed with SMRT (v.9.0.0, Pacific Biosciences). Primer removal, quality filtering (maximum expected errors 2, minimum length 500 bp, discarded reads that match phiX), truncation (after 1,800 bp or at the first instance of a quality score <3), dereplication, denoising and chimaera removal were carried out using the R package dada2 (v.3.10)^74^. ASVs (amplicon sequence variants) were clustered by 97% similarity using the R package DECIPHER (v.2.16.1)^75^. A Bayesian classifier was used to assign the taxonomy with the UTAX reference dataset (utax_reference_dataset_10.05.2021.fasta) from the UNITE database^76^.

### Analysis of soil microbiome predictors

The OTU and taxonomy tables as well as the sample data were imported into the R package phyloseq (v.1.36.0)^77^. OTUs with zero counts were removed and the four replicates of each sample were merged and the counts summed. All samples were rarefied to an even sampling depth using the smallest sample number (that is, 4,272 reads). The resulting OTU table can be found in Supplementary Table 6. Rarefaction curves (rarecurve function in the R package vegan v.2.5-7)^78^ were calculated to determine sufficient sampling depth.

Spearman rank correlations (corr.test function of the R package psych v.2.1.9)^67^ were calculated for individual OTUs and MGR, and corrected for multiple testing using the Benjamini–Hochberg procedure^68^.

To filter the OTU table to a more reduced candidate set for model selection, three independent analyses were performed. An indicator species analysis was performed with low- and high-MGR fields (multipatt function in the R package indicspecies (v.1.7.9), function ‘r.g’, 999 permutations)^79^. Indicator OTUs with *P* < 0.1 were retained. A less stringent significance threshold of 0.1 was chosen to obtain a larger candidate set of 29 OTUs (Supplementary Table 7). Differential abundance of OTUs between low- and high-MGR fields was assessed using DESeq2 (v.1.30.1)^80^, and the Wald significance tests and parametric fitting. Using a significance threshold of 0.1 resulted in the selection of 18 OTUs (Supplementary Table 8). Random forest analysis (R package randomForest v.4.6-14)^29^ was performed to identify the most important predictors of MGR. OTUs with IncNodePurity >30 are listed in Supplementary Table 9. The combined results of these analyses produced a subset of 44 OTUs. For final model selection in combination with soil parameters, this subset was further reduced using glmulti (v.1.0.8)^31^ with exhaustive screening of all possible models and the Akaike information criterion with correction for small sample sizes (aicc).

Automatic species assignment against the reference database often did not result in annotations at lower taxonomic levels. Therefore, the most important OTUs were additionally subjected to a BLAST search against the NCBI database (Supplementary Table 10).

Principal coordinate analysis (PCoA) was performed on the square-root-transformed OTUs on the basis of Bray–Curtis dissimilarities (vegdist function in the R package vegan v.2.5-7)^78^ to investigate a possible year effect (Extended Data Fig. 3). Subsequently, to explore relationships between soil fungal community composition and MGR, partial distance-based redundancy analysis (dbRDA) was performed using the capscale function in the R package vegan (v.2.5-7)^78^, with the variable ‘Year’ as the condition that was partialled out. The loading vectors corresponding to the final set of 13 soil OTUs selected by the methods described above were added to the partial dbRDA plot (Fig. 3b).

FUNGuild (v1.2)^81^ was used to assess fungal guilds and group pathogen sOTUs (Supplementary Table 13).

### Linear regression models

The lm function of the R package MASS (v.7.3-54)^30^ was used to develop linear regression models. Violation of normality assumption was assessed using Ols_test_normality in the R package olsrr (v.0.5.3)^82^. The relative importance of each predictor in the models was evaluated using the package relaimpo (v.2.2-6)^83^ (Fig. 5). For the correlation plots of summed high- and low-MGR OTUs and MGR, a linear regression model was developed using only the 15 soil parameters. Residuals were extracted using the residual function in the R package stats (v.4.0.3)^63^ and plotted against OTU abundance.

### Cross-validation of models

To cross-validate our models, we split the dataset into a training dataset (90% of the dataset) and a test dataset (10%) using the createDataPartition function of the R package caret (v.6.0-94)^84^. Since the ultimate goal is not to predict the exact value of MGR, a binary classification approach was chosen to test the accuracy of the model in predicting inoculation success. An MGR of 12.2% was chosen since this represented the lower limit for significant positive effects (‘yes’: positive growth effect, >12.2% MGR; ‘no’: neutral or negative growth effect, <12.2% MGR). The linear regression models were developed as described above using the test dataset. MGR values of the test dataset were predicted using the predict function of the R package car (v.3.1.0)^85^. The number of true positive, true negative, false positive and false negative predictions were assessed using the confusionMatrix function of the R package car (v.3.1.0). The mean accuracy was assessed over 1,000 iterations (randomly splitting the dataset into training and test data).

### Root microbiome analysis

The OTU and taxonomy tables as well as sample data were imported into the R package phyloseq (v.1.36.0)^77^. OTUs with zero counts were removed. All samples were subsampled to an even sampling depth using the smallest sample number (that is, 1,660). The relative abundances at the phylum level were assessed and visualized in a bar chart (Supplementary Fig. 3). Rarefaction curves (rarecurve function in the R package vegan v.2.5-7)^78^ were calculated to assess sufficient sampling depth (Supplementary Fig. 2). The rarefied root OTU table can be found in Supplementary Table 11. For the visualization of the community composition at the genus level, the 15 most abundant genera were selected from the rarefied OTU table (Supplementary Fig. 3).

To investigate similarities of the native root microbiome between the MGR categories (that is, low, medium, high) and a possible year effect, control samples were selected from the OTU table. First, PCoA (cmdscale function in the R package stats (v.4.0.3))^63^ was performed on the root-transformed OTUs and on the basis of the Bray–Curtis dissimilarities (vegdist function in the R package vegan v.2.5-7)^78^ and samples were coloured by year. Subsequently, partial dbRDA was performed using the capscale function in the R package vegan (v.2.5-7)^78^, with the variable ‘Year’ as the condition that was partialled out (Extended Data Fig. 6).

**Extended Data Fig. 9:**
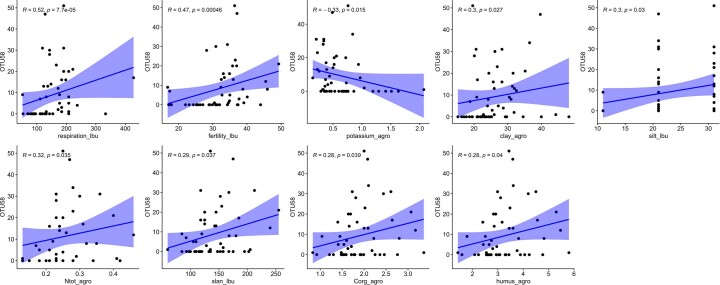
Scatter plots of significant correlations of soil physico-chemical variables with the most important indicator OTU for low MGR. OTU58 (no database match) correlates significantly with properties indicative of healthy soils (that is, high respiration, fertility and organic carbon). The correlation coefficients (Spearman rank) and the significant values (p) are displayed in the plots. The blue shaded area represents the 95% confidence interval.

**Extended Data Fig. 10:**
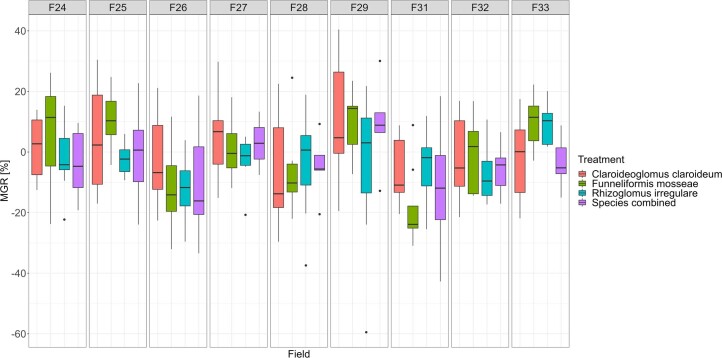
Inoculation trial with different AMF species. Individual and combined inoculations of different AMF species did not result in any significant differences in MGR between species (p = 0.2006, F = 1.51) or significant interactions between fields and different species (p = 0.4124, F = 1.04,), as revealed by a 2-wy ANOVA (n = 8 individual plots per treatment group). The boxes represent the interquartile range (IQR, 25th to 75th percentile). The horizontal line within the box represents the median (50th percentile). The whiskers represent the minima and maxima of the data in comparison to the IQR (±1.5*IQR). Outliers are represented as individual data points.

To evaluate the establishment success of the inoculated SAF22, PCoA was performed on control and inoculated samples, and results were coloured according to these two treatment categories (Extended Data Fig. 9). Further, OTUs corresponding to SAF22 (rOTU2, rOTU4, rOTU9, rOTU16, rOTU74, rOTU84, rOTU165) were summed and their relative abundances recorded for control and inoculated samples, as well as their differences (Fig. 3c and Supplementary Table 12).

To investigate whether shifts in community structure differed between low- and high-MGR fields, we performed a differential abundance analysis (that is, control vs inoculated) for low- and high-MGR fields separately. We used the R package DESeq2 (v.1.30.1)^80^, Wald significance tests, parametric fitting and a significance threshold of 0.1 (Fig. 6, and Supplementary Tables 14 and 15).

### Root colonization

Total root colonization by AMF was assessed using the magnified section method^86^. First, roots were cleared with KOH and stained with an ink–vinegar mixture^87^. Approximately 30 cm of roots, consisting of 1–2-cm-long pieces, were mounted on a microscope slide and 100 intersections per sample were counted. The intersection types included ‘negative’, ‘arbuscule’, ‘vesicle’ and ‘internal hypha’. Total root colonization was recorded as a percentage of all non-negative intersections (Fig. 3c and Supplementary Table 12).

### Reporting summary

Further information on research design is available in the Nature Portfolio Reporting Summary linked to this article.

### Supplementary information

Supplementary Information Supplementary Figs. 1–5; Tables 2–5, 7–10, 12, 14–16; and Results and Discussion.

Reporting Summary

Supplementary Tables Supplementary Tables 1, 6, 11, 13.

## Data Availability

The raw sequencing data are stored in the European Nucleotide Archive (http://www.ebi.ac.uk/ena) under accession numbers PRJEB53587 (soil microbiome) and PRJEB56590 (root microbiome). All other data are available in the supplementary material.

## Code availability

All code and input files are available at https://github.com/PMI-Basel/Lutz_et_al_Predicting_crop_yield.

## References

[1] Foley JA (2011). Solutions for a cultivated planet. Nature.

[2] Foley JA (2005). Global consequences of land use. Science.

[3] *The State of Food Security and Nutrition in the World 2022* (FAO, 2022).

[4] Bender SF, Wagg C, van der Heijden MGA (2016). An underground revolution: biodiversity and soil ecological engineering for agricultural sustainability. Trends Ecol. Evol..

[5] Banerjee S, van der Heijden MGA (2022). Soil microbiomes and one health. Nat. Rev. Microbiol..

[6] Trivedi P, Leach JE, Tringe SG, Sa T, Singh BK (2020). Plant–microbiome interactions: from community assembly to plant health. Nat. Rev. Microbiol..

[7] Trivedi P, Batista BD, Bazany KE, Singh BK (2022). Plant–microbiome interactions under a changing world: responses, consequences and perspectives. New Phytol..

[8] Walder F, van der Heijden MGA (2015). Regulation of resource exchange in the arbuscular mycorrhizal symbiosis. Nat. Plants.

[9] Lekberg Y, Koide RT (2005). Is plant performance limited by abundance of arbuscular mycorrhizal fungi? A meta-analysis of studies published between 1988 and 2003. New Phytol..

[10] Hoeksema JD (2010). A meta-analysis of context-dependency in plant response to inoculation with mycorrhizal fungi. Ecol. Lett..

[11] Chaudhary VB (2016). MycoDB, a global database of plant response to mycorrhizal fungi. Sci. Data.

[12] Watts-Williams SJ, Cavagnaro TR (2014). Nutrient interactions and arbuscular mycorrhizas: a meta-analysis of a mycorrhiza-defective mutant and wild-type tomato genotype pair. Plant Soil.

[13] Cavagnaro TR, Bender SF, Asghari HR, van der Heijden MGA (2015). The role of arbuscular mycorrhizas in reducing soil nutrient loss. Trends Plant Sci..

[14] Bender SF (2013). Symbiotic relationships between soil fungi and plants reduce N_2_O emissions from soil. ISME J..

[15] Zhang X, Wang L, Ma F, Shan D (2015). Effects of arbuscular mycorrhizal fungi on N_2_O emissions from rice paddies. Water Air Soil Pollut..

[16] Begum N (2019). Improved drought tolerance by AMF inoculation in maize (*Zea mays*) involves physiological and biochemical implications. Plants.

[17] Augé RM, Kubikova E, Moore JL (2001). Foliar dehydration tolerance of mycorrhizal cowpea, soybean and bush bean. New Phytol..

[18] Newsham KK, Fitter AH, Watkinson AR (1995). Arbuscular mycorrhiza protect an annual grass from root pathogenic fungi in the field. J. Ecol..

[19] Azcón-Aguilar, C., Jaizme-Vega, M. C. & Calvet, C. in *Mycorrhizal Technology in Agriculture* (eds Gianinazzi, S. et al.) 187–197 (Springer, 2002).

[20] Jansa J (2002). Diversity and structure of AMF communities as affected by tillage in a temperate soil. Mycorrhiza.

[21] Verbruggen E (2010). Positive effects of organic farming on below-ground mutualists: large-scale comparison of mycorrhizal fungal communities in agricultural soils. New Phytol..

[22] Loit K (2018). The indigenous arbuscular mycorrhizal fungal colonisation potential in potato roots is affected by agricultural treatments. Agron. Res..

[23] Zhang S, Lehmann A, Zheng W, You Z, Rillig MC (2019). Arbuscular mycorrhizal fungi increase grain yields: a meta-analysis. New Phytol..

[24] Köhl L, Lukasiewicz CE, van der Heijden MGA (2016). Establishment and effectiveness of inoculated arbuscular mycorrhizal fungi in agricultural soils. Plant Cell Environ..

[25] Klironomos JN (2003). Variation in plant response to native and exotic arbuscular mycorrhizal fungi. Ecology.

[26] van der Heijden, M. G. A. in *Mycorrhizal Ecology* Ecological Studies Vol 157 (eds van der Heijden, M. G. A. & Sanders, I. R.) 243–265 (Springer, 2002).

[27] Salomon MJ (2022). Global evaluation of commercial arbuscular mycorrhizal inoculants under greenhouse and field conditions. Appl. Soil Ecol..

[28] Bodenhausen N (2023). Predicting soil fungal communities from chemical and physical properties. J. Sustain. Agric. Environ..

[29] Liaw A, Wiener M (2002). Classification and regression by randomForest. R News.

[30] Ripley, W. N. & Venables, B. D. *Modern Applied Statistics with S* (Springer, 2002).

[31] Calcagno, V. & de Mazancourt, C. glmulti: an R package for easy automated model selection with (generalized) linear models. *J. Stat. Softw.*10.18637/jss.v034.i12 (2010).

[32] Sinclair TR (2011). Challenges in breeding for yield increase for drought. Trends Plant Sci..

[33] Wittwer RA, Dorn B, Jossi W, van der Heijden MGA (2017). Cover crops support ecological intensification of arable cropping systems. Sci. Rep..

[34] Schmidt, J. E. & Gaudin, A. C. M. What is the agronomic potential of biofertilizers for maize? A meta-analysis. *FEMS Microbiol. Ecol.*10.1093/femsec/fiy094 (2018). 10.1093/femsec/fiy094 29796593

[35] Bender SF, Schlaeppi K, Held A, van der Heijden MGA (2019). Establishment success and crop growth effects of an arbuscular mycorrhizal fungus inoculated into Swiss corn fields. Agric. Ecosyst. Environ..

[36] Hirte J, Richner W, Orth B, Liebisch F, Flisch R (2021). Yield response to soil test phosphorus in Switzerland: pedoclimatic drivers of critical concentrations for optimal crop yields using multilevel modelling. Sci. Total Environ..

[37] Ngatia (2021). Land use change affects soil organic carbon: an indicator of soil health. Environ. Health.

[38] Toda M, Walder F, van der Heijden MGA (2023). Organic management and soil health promote nutrient use efficiency. J. Sustain. Agric. Environ..

[39] Bonanomi G, Antignani V, Capodilupo M, Scala F (2010). Identifying the characteristics of organic soil amendments that suppress soilborne plant diseases. Soil Biol. Biochem..

[40] Vida C, de Vicente A, Cazorla FM (2020). The role of organic amendments to soil for crop protection: induction of suppression of soilborne pathogens. Ann. Appl. Biol..

[41] Mozafar A, Anken T, Ruh R, Frossard E (2000). Tillage intensity, mycorrhizal and nonmycorrhizal fungi, and nutrient concentrations in maize, wheat, and canola. Agron. J..

[42] Agrios, G. N. *Plant Pathology* 5th edn (Elsevier, 2005).

[43] Saira M (2017). First report of *Myrothecium verrucaria* causing leaf spot of maize in Pakistan. Plant Dis..

[44] Mesterházy Á, Lemmens M, Reid LM (2012). Breeding for resistance to ear rots caused by *Fusarium* spp. in maize – a review. Plant Breed..

[45] Goswami RS, Kistler HC (2004). Heading for disaster: *Fusarium graminearum* on cereal crops. Mol. Plant Pathol..

[46] Colombo AL, Padovan ACB, Chaves GM (2011). Current knowledge of *Trichosporon* spp. and trichosporonosis. Clin. Microbiol. Rev..

[47] Hart MM, Antunes PM, Chaudhary VB, Abbott LK (2018). Fungal inoculants in the field: is the reward greater than the risk?. Funct. Ecol..

[48] van der Heijden MGA (2006). The mycorrhizal contribution to plant productivity, plant nutrition and soil structure in experimental grassland. New Phytol..

[49] Cordier, C., Pozo, M. J., Barea, J. M., Gianinazzi, S. & Gianinazzi-Pearson, V. Cell defense responses associated with localized and systemic resistance to *Phytophthora parasitica* induced in tomato by an arbuscular mycorrhizal fungus. *Mol. Plant Microbe Interact.* **11**, 1017–1028 (2007).

[50] Lareen A, Burton F, Schäfer P (2016). Plant root-microbe communication in shaping root microbiomes. Plant Mol. Biol..

[51] Whipps, J. M. Prospects and limitations for mycorrhizas in biocontrol of root pathogens. *Can. J. Bot.* **82**, 1198–1227 (2004).

[52] Schlaeppi K (2016). High-resolution community profiling of arbuscular mycorrhizal fungi. New Phytol..

[53] Gerlach N (2015). An integrated functional approach to dissect systemic responses in maize to arbuscular mycorrhizal symbiosis. Plant Cell Environ..

[54] Pozo MJ, Azcón-Aguilar C (2007). Unraveling mycorrhiza-induced resistance. Curr. Opin. Plant Biol..

[55] Pieterse, C. M. J. et al. Induced systemic resistance by beneficial microbes. *Annu. Rev. Phytopathol.* **52**, 347–375, (2014). 10.1146/annurev-phyto-082712-102340 24906124

[56] Svenningsen NB (2018). Suppression of the activity of arbuscular mycorrhizal fungi by the soil microbiota. ISME J..

[57] Riedo J (2021). Widespread occurrence of pesticides in organically managed agricultural soils – the ghost of a conventional agricultural past?. Environ. Sci. Technol..

[58] Edlinger A (2022). Agricultural management and pesticide use reduce the functioning of beneficial plant symbionts. Nat. Ecol. Evol..

[59] Tedersoo L, Albertsen M, Anslan S, Callahan B (2021). Perspectives and benefits of high-throughput long-read sequencing in microbial ecology. Appl. Environ. Microbiol..

[60] Sawers RJH (2017). Phosphorus acquisition efficiency in arbuscular mycorrhizal maize is correlated with the abundance of root-external hyphae and the accumulation of transcripts encoding PHT1 phosphate transporters. New Phytol..

[61] Chowdhury SP, Hartmann A, Gao XW, Borriss R (2015). Biocontrol mechanism by root-associated *Bacillus amyloliquefaciens* FZB42 – a review. Front. Microbiol..

[62] Richner, W., Bretscher, D., Menzi, H. & Prasuhn, V. Grundlagen für die Düngung landwirtschaftlicher Kulturen in der Schweiz. *Agrarforsch. Schweiz* **8**, 1–12 (2017).

[63] R Core Team. *R: A Language and Environment for Statistical Computing* (R Foundation for Statistical Computing, 2020).

[64] Villanueva, R. A. M. & Chen, Z. J. ggplot2: elegant graphics for data analysis (2nd edn). *Measurement* **17**, 160–167 (2019).

[65] Kassambara, A. ggpubr:’ggplot2’ based publication ready plots. R package version 3.3.5 https://cran.r-project.org/package=ggpubr (2020).

[66] Bah, T. *Inkscape Guide to a Vector Drawing Program* 3rd edn (Prentice Hall Press, 2011).

[67] Revelle, W. *Procedures for Personality and Psychological Research* (Northwestern Univ., 2015).

[68] Benjamini Y, Hochberg Y (1995). Controlling the false discovery rate: a practical and powerful approach to multiple testing. J. R. Stat. Soc. B.

[69] Wickham, H. Reshaping data with the reshape package. *J. Stat. Softw.*10.18637/jss.v021.i12 (2007).

[70] Gardes M, Bruns TD (1993). ITS primers with enhanced specificity for basidiomycetes-application to the identification of mycorrhizae and rusts. Mol. Ecol..

[71] White, T. J., Bruns, T., Lee, S. & Taylor, J. in *PCR Protocols* (eds Innis, M. A. et al.) 315–322 (Elsevier, 1990); 10.1016/B978-0-12-372180-8.50042-1

[72] Bodenhausen N (2019). *Petunia*- and *Arabidopsis*-specific root microbiota responses to phosphate supplementation. Phytobiomes J..

[73] Krüger M, Krüger C, Walker C, Stockinger H, Schüßler A (2012). Phylogenetic reference data for systematics and phylotaxonomy of arbuscular mycorrhizal fungi from phylum to species level. New Phytol..

[74] Benjamin J (2016). DADA2: high-resolution sample inference from Illumina amplicon data. Nat. Methods.

[75] Wright, E. S. Using DECIPHER v2.0 to analyze big biological sequence data in R. *R J.* **8**, 352–359 (2016).

[76] Nilsson RH (2019). The UNITE database for molecular identification of fungi: handling dark taxa and parallel taxonomic classifications. Nucleic Acids Res..

[77] McMurdie PJ, Holmes S (2013). phyloseq: an R package for reproducible interactive analysis and graphics of microbiome census data. PLoS ONE.

[78] Oksanen, J. et al. vegan: An R package for community ecologists. R package version 2.5-7 https://cran.r-project.org/package=vegan (2020).

[79] De Cáceres M, Legendre P (2009). Associations between species and groups of sites: indices and statistical inference. Ecology.

[80] Love MI, Huber W, Anders S (2014). Moderated estimation of fold change and dispersion for RNA-seq data with DESeq2. Genome Biol..

[81] Nguyen NH (2016). FUNGuild: an open annotation tool for parsing fungal community datasets by ecological guild. Fungal Ecol..

[82] Hebbali, A. olsrr: Tools for building OLS regression models. R package version 0.5.3 https://cran.r-project.org/package=olsrr (2020).

[83] Grömping, U. Relative importance for linear regression in R: the package relaimpo. *J. Stat. Softw.*10.18637/jss.v017.i01 (2007).

[84] Hyndman, R. J. & Athanasopoulos, G. *Forecasting: Principles and Practice* Ch. 3.1 (OTexts, 2013).

[85] Fox, J. & Weisberg, S. *An R Companion to Applied Regression* (SAGE Publications, 2018).

[86] McGonigle TP, Miller MH, Evans DG, Fairchild GL, Swan JA (1990). A new method which gives an objective measure of colonization of roots by vesicular–arbuscular mycorrhizal fungi. New Phytol..

[87] Vierheilig H, Coughlan AP, Wyss U, Piché Y (1998). Ink and vinegar, a simple staining technique for arbuscular–mycorrhizal fungi. Appl. Environ. Microbiol..

