# Author Correction: Soil microbiome indicators can predict crop growth response to large-scale inoculation with arbuscular mycorrhizal fungi

**DOI:** 10.1038/s41564-023-01577-7

**Published:** 2023-12-19

**Authors:** Stefanie Lutz, Natacha Bodenhausen, Julia Hess, Alain Valzano-Held, Jan Waelchli, Gabriel Deslandes-Hérold, Klaus Schlaeppi, Marcel G. A. van der Heijden

**Affiliations:** 1 Plant–Soil Interactions, Department of Agroecology and Environment, Agroscope, Zurich, Switzerland; 2 Department of Soil Sciences, Research Institute of Organic Agriculture (FiBL), Frick, Switzerland; 3 Plant Microbe Interactions, Department of Environmental Sciences, University of Basel, Basel, Switzerland; 4 Institute of Plant Sciences, University of Bern, Bern, Switzerland; 5 Department of Plant and Microbial Biology, University of Zürich, Zurich, Switzerland; 6 Present Address: Plant Biochemistry, Institute of Molecular Plant Biology, ETH Zurich, Zurich, Switzerland

**Keywords:** Physiology, Diseases, Microbiome, Arbuscular mycorrhiza

Correction to: *Nature Microbiology* 10.1038/s41564-023-01520-w, published online 29 November 2023.

This article was originally published under a standard Springer Nature licence (© The Author(s), under exclusive licence to Springer Nature Limited). It is now available as an open-access paper under a Creative Commons Attribution 4.0 International licence, © The Author(s). The error has been corrected in the HTML and PDF versions of the article.

