# Supplementary material for: Soil microbiome indicators can predict crop growth response to large-scale inoculation with arbuscular mycorrhizal fungi
Source: Nat Microbiol. 2023 Nov 29;8(12):2277–89. doi: 10.1038/s41564-023-01520-w (PMC10730404; doi:10.1038/s41564-023-01520-w)
Supplement: Supplementary file 1 — Supplementary Figs. 1–5; Tables 2–5, 7–10, 12, 14–16; and Results and Discussion. [file 41564_2023_1520_MOESM1_ESM.pdf]

nature microbiology

Article https://doi.org/10.1038/s41564-023-01520-w

Soil microbiome indicators can predict crop growth response to large-scale inoculation with arbuscular mycorrhizal fungi

In the format provided by the authors and unedited

Table of contents:

Supplementary Results and Discussion 2
Most important soil parameters for MGR prediction 2
Model with all fungal sOTUs classified as pathogens not able to predict MGR 2
Root microbiome data confirms findings of MGR prediction model 3
Supplementary Fig. 1: Relative abundance of the root microbiome at the phylum level. 4
Supplementary Fig. 2: Rarefaction curves of subsampled root OTU table. 5
Supplementary Fig. 3 A (2018): Root community composition at genus level 6
Supplementary Fig. 3 B (2019): Root community composition at genus level 7
Supplementary Fig. 3 C (2020): Root community composition at genus level 8
Supplementary Fig. 4: Relationship between all pathogen sOTUs and MGR. 9
Supplementary Fig. 5: Correlation of plant biomass and yield data. 10
Supplementary Table 1: Overview of inoculated fields including corresponding mycorrhizal growth response and soil parameters. 11
Supplementary Table 2: Pairwise correlations of soil parameters and MGR. 12
Supplementary Table 3: Overview of co-correlated parameters. 14
Supplementary Table 4: Random forest analysis of soil parameters. 15
Supplementary Table 5: Soil parameters selected for final model input. 16
Supplementary Table 6: Soil OTU table. 17
Supplementary Table 7: Results of the indicator species analysis of fields with low and high MGR. 18
Supplementary Table 8: Results of the DESeq2 analysis of soil OTUs. 20
Supplementary Table 9: Random forest analysis of soil OTUs. 21
Supplementary Table 10: Soil fungal OTUs selected for final model input. 22
Supplementary Table 11: Root OTU table. 25
Supplementary Table 12: AMF establishment success. 26
Supplementary Table 13: Fungal guilds of soil OTUs. 28
Supplementary Table 14: Results of the DESeq2 analysis of root OTUs in low MGR fields. ...29
Supplementary Table 15: Results of the DESeq2 analysis of root OTUs in high MGR fields...31

Supplementary Results and Discussion

Most important soil parameters for MGR prediction (extended)

The 15 soil parameters in the full model could explain about 26 % of the variations in MGR (**Fig. 4 A**). These comprised cMIC, Nmin and magnesium. Total nitrogen content was negatively correlated with MGR (**Supplementary Table 2**), while SAF22 was positively associated with MGR in the model. A higher content of easily leachable magnesium (H2O extraction) was also associated with a higher MGR, while this was reversed for more metal-bound and thus less bioavailable magnesium (EDTA extraction). This could be due to the fact that magnesium plays an important role in stimulating root colonisation by AMF (Gryndler, Vejsadová, and Vančura 1992) when bioavailable.

Most important soil OTUs for MGR prediction (extended)

Analogous to the soil parameters we reduced the number of candidate sOTUs for model input, again using different approaches (**Fig. 3 B**). An indicator species analysis revealed 11 and 20 sOTUs characteristic of fields with high and low MGR, respectively (**Supplementary Table 7**). Differential abundance analysis between fields with high and low MGR resulted in 5 and 14 sOTUs, respectively (**Supplementary Table 8**). Furthermore, a random forest analysis was performed on the continuous MGR values (**Supplementary Table 9**). Only the top 30 predictors were retained since this number of sOTUs corresponds to the identified number of sOTUs in the other two analyses.

Comparing all three methods (**Supplementary Table 10**), only two fungal sOTUs were identified as important by all three methods (sOTU18: *Trichosporon* sp. (high MGR) and sOTU182: *Dendryphion europaeum* (low MGR)), which was in contrast to the relatively large overlap of soil parameters selected by different approaches. In addition, seven sOTUs were selected by two methods and 49 sOTUs by one method only. For reasons of stricter selectivity and due to the small overlap between the random forest and the other two analyses, sOTUs identified solely by random forest were not retained. The combined results of the indicator species and DESeq2 analyses yielded a total of 44 sOTUs.

While fields with high MGR were characterized by higher pathogen load, the main indicators of low MGR included the genera *Phaeohelotium*, *Phaeosphaeria*, *Powellomyces* and three sOTUs without database match. Not much is known about these genera in the literature, but they could be indicators of generally healthy soils, as suggested by the positive correlation of the most important predictor OTU58 (no database match) with soil fertility and microbial respiration (**Supplementary Fig. 13**).

Model with all fungal sOTUs classified as pathogens not able to predict MGR

Since soil pathogenic fungi were the strongest predictors in the model, we investigated whether the relative abundance of all soil pathogenic fungi would perform better in predicting MGR. Therefore, we evaluated fungal guilds of all sOTUs using FUNGuild and summed the abundance of sOTUs that were classified as pathogens (Supplementary Table 13). However, their abundance was neither significantly correlated with MGR (R=0.029, p=0.83; Supplementary Fig. 20) nor performed well in a linear regression model (R= 0.0036, p= 0.6701). On the one hand, this could be because in most cases there are no exact matches in the sequence databases and because even within a species, traits are often highly diverse. Therefore, functional annotations are often unreliable if not conducted on the strain level. On the other hand, it may indeed be that only a few pathogens AMF strains are likely specialised in their ability to protect against specific pathogens.

Root microbiome data confirms findings of MGR prediction model

Of note, we identified *Trichosporon*, the most important soil fungal OTU in the predictive model, also in the data of the root fungal communities (rOTU20 shares 100% sequence similarity with sOTU18 over its entire amplicon length). Although the *Trichosporon* rOTU20 was not significantly (p=0.263) different in abundance between control or inoculated roots, it showed a trend of a decrease (log2FC=-0.583) similar to the differentially abundant pathogens in roots of fields with high MGR (**Supplementary Table 15**).

Inoculum application

The inoculum was manually mixed with field soils. This application mode is highly suitable for scientific experiments, but it is not feasible for large scale agricultural production. In order to achieve a better cost-benefit ratio, the dosage and method of application of the AMF inoculum must also be optimised, ideally in cooperation with farmers and specialised biotech companies.

References

Bender, S. Franz, Klaus Schlaeppi, Alain Held, and Marcel G.A. Van der Heijden. 2019. "Establishment Success and Crop Growth Effects of an Arbuscular Mycorrhizal Fungus Inoculated into Swiss Corn Fields." *Agriculture, Ecosystems & Environment* 273 (March): 13–24. https://doi.org/10.1016/J.AGEE.2018.12.003.

Gryndler, M., H. Vejsadová, and V. Vančura. 1992. "The Effect of Magnesium Ions on the Vesicular—Arbuscular Mycorrhizal Infection of Maize Roots." *New Phytologist* 122 (3): 455–60. https://doi.org/10.1111/J.1469-8137.1992.TB00073.X.

Supplementary Fig. 1: Relative abundance of the root microbiome at the phylum level.

Control samples are shown on the left and corresponding samples inoculated with the AMF SAF22 on the right. Sequencing of the root microbiome with AMF-specific primers led not not only to the amplification of the main target phylum Glomeromycota, but also Ascomycota and Basidiomycota. The plot is based on the rarefied OTU table. White bars represent missing samples that could not be sequenced due to the concentrations of the PCR products being too low.

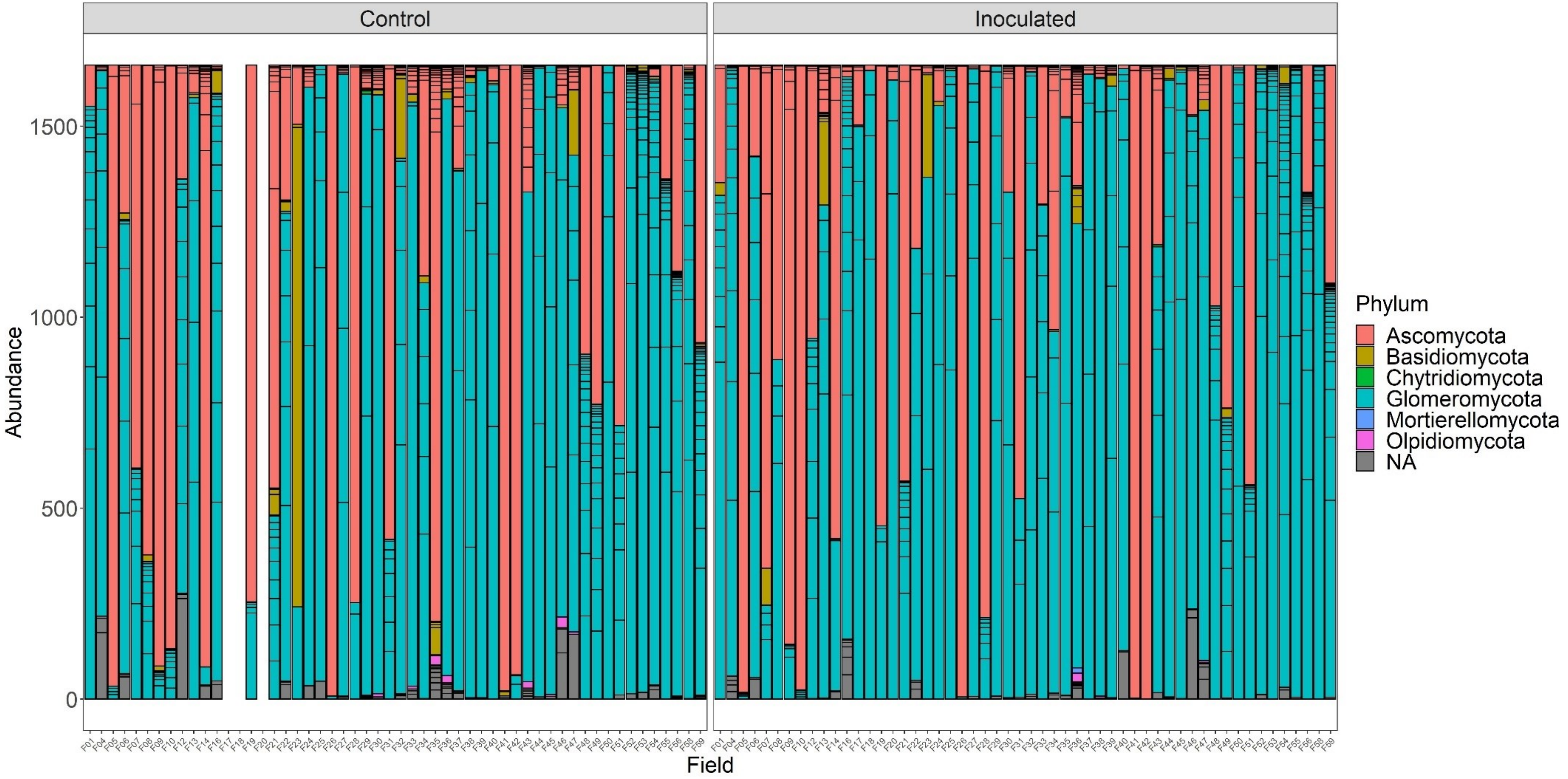

Supplementary Fig. 2: Rarefaction curves of subsampled root OTU table.

Plateauing curves show that sufficient sequencing depth was retained after rarefying to an even sequence number of 1660 reads per sample.

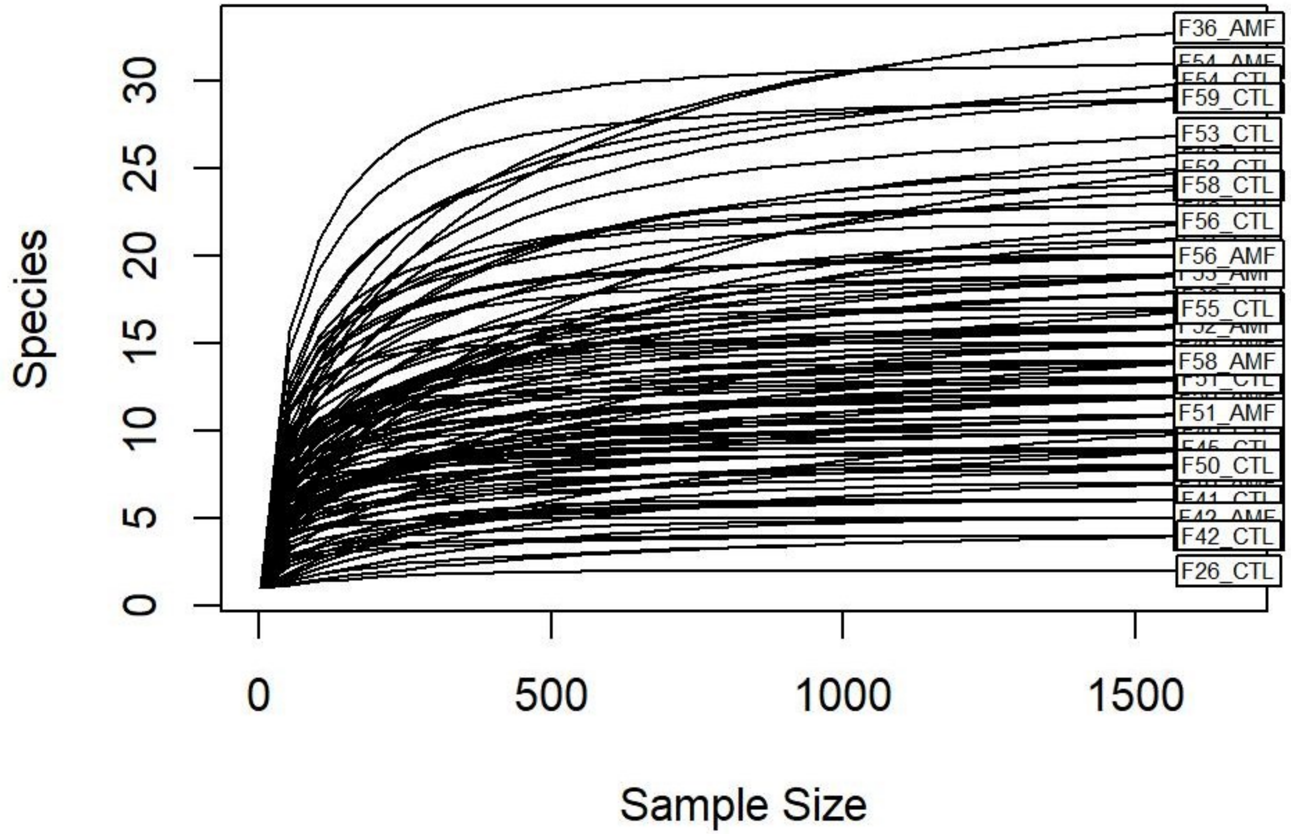

Supplementary Fig. 3 A (2018): Root community composition at genus level.

The plot shows the relative abundance of the root microbiome for control (C) and inoculated (I) samples for each field. The 15 most abundant genera were selected for better visualisation. Within these genera, OTUs without genus assignment are summarised in grey (genus not assigned). Less abundant genera are displayed in black. The establishment success of the inoculated *Rhizoglomus irregulare* SAF22 is shown below the field IDs and represents the difference between inoculated and control samples. White bars represent missing samples that could not be sequenced due to the concentrations of the PCR products being too low.

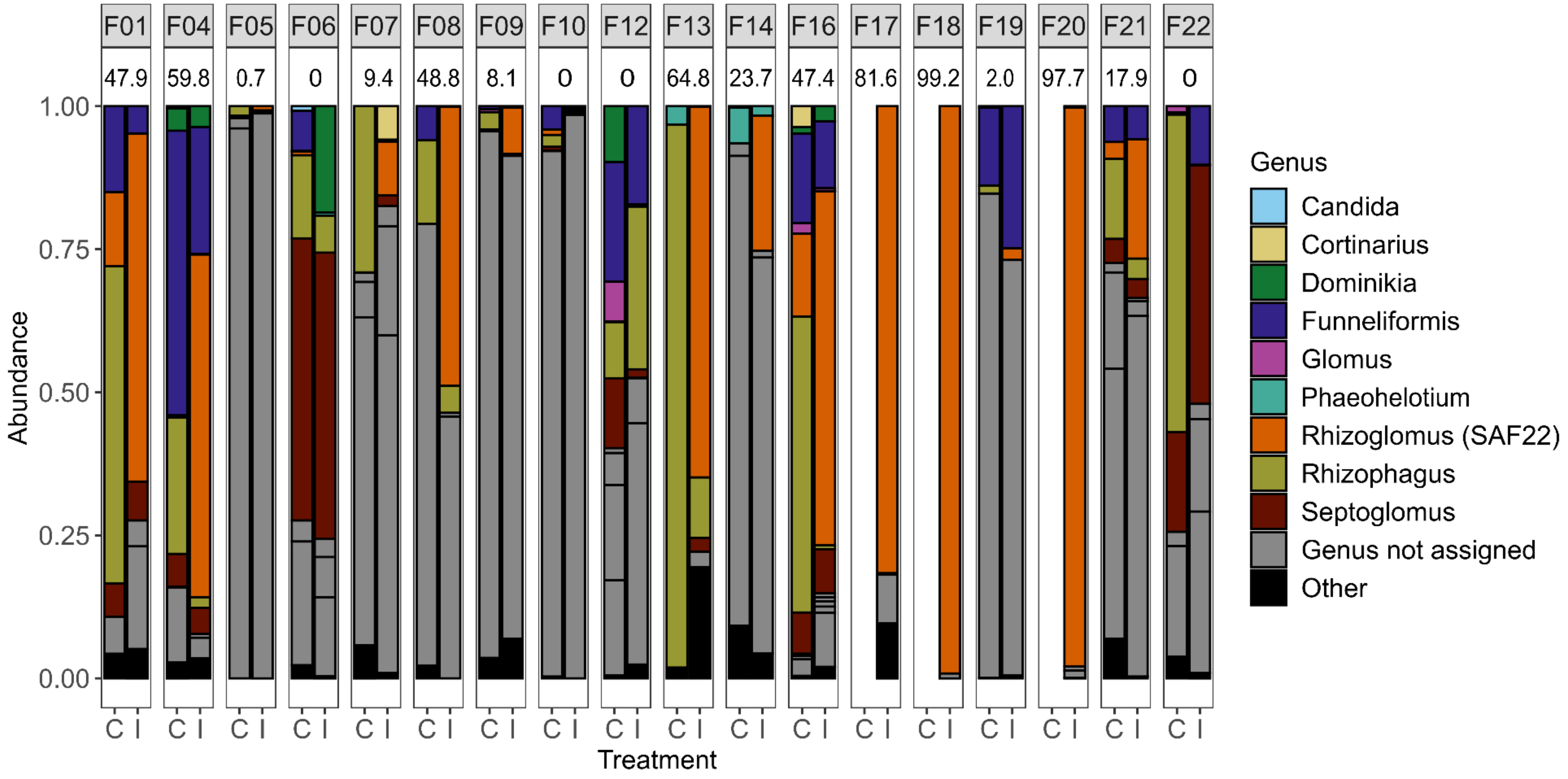

Supplementary Fig. 3 B (2019): Root community composition at genus level.

The plot shows the relative abundance of the root microbiome for control (C) and inoculated (I) samples for each field. The 15 most abundant genera were selected for better visualisation. Within these genera, OTUs without genus assignment are summarised in grey (genus not assigned). Less abundant genera are displayed in black. The establishment success of the inoculated *Rhizoglomus irregulare* SAF22 is shown below the field IDs and represents the difference between inoculated and control samples. White bars represent missing samples that could not be sequenced due to the concentrations of the PCR products being too low.

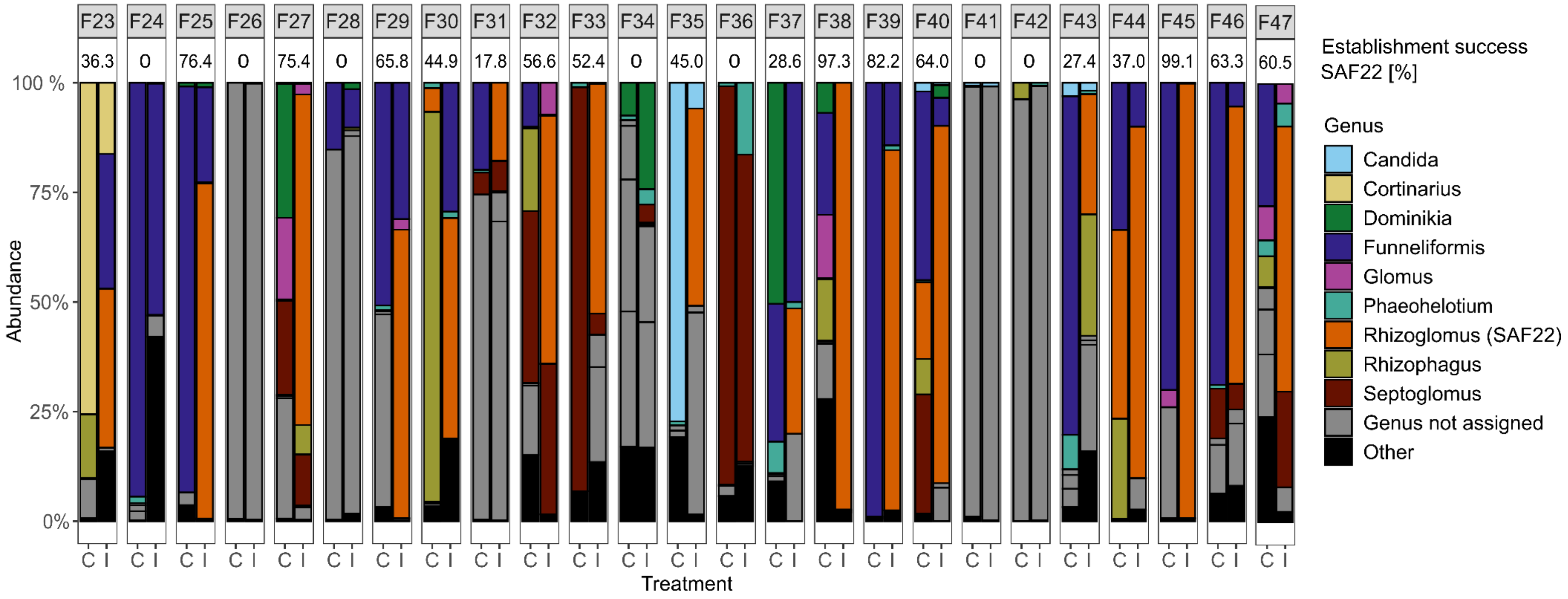

Supplementary Fig. 3 C (2020): Root community composition at genus level.

The plot shows the relative abundance of the root microbiome for control (C) and inoculated (I) samples for each field. The 15 most abundant genera were selected for better visualisation. Within these genera, OTUs without genus assignment are summarised in grey (genus not assigned). Less abundant genera are displayed in black. The establishment success of the inoculated *Rhizoglomus irregulare* SAF22 is shown below the field IDs and represents the difference between inoculated and control samples. White bars represent missing samples that could not be sequenced due to the concentrations of the PCR products being too low.

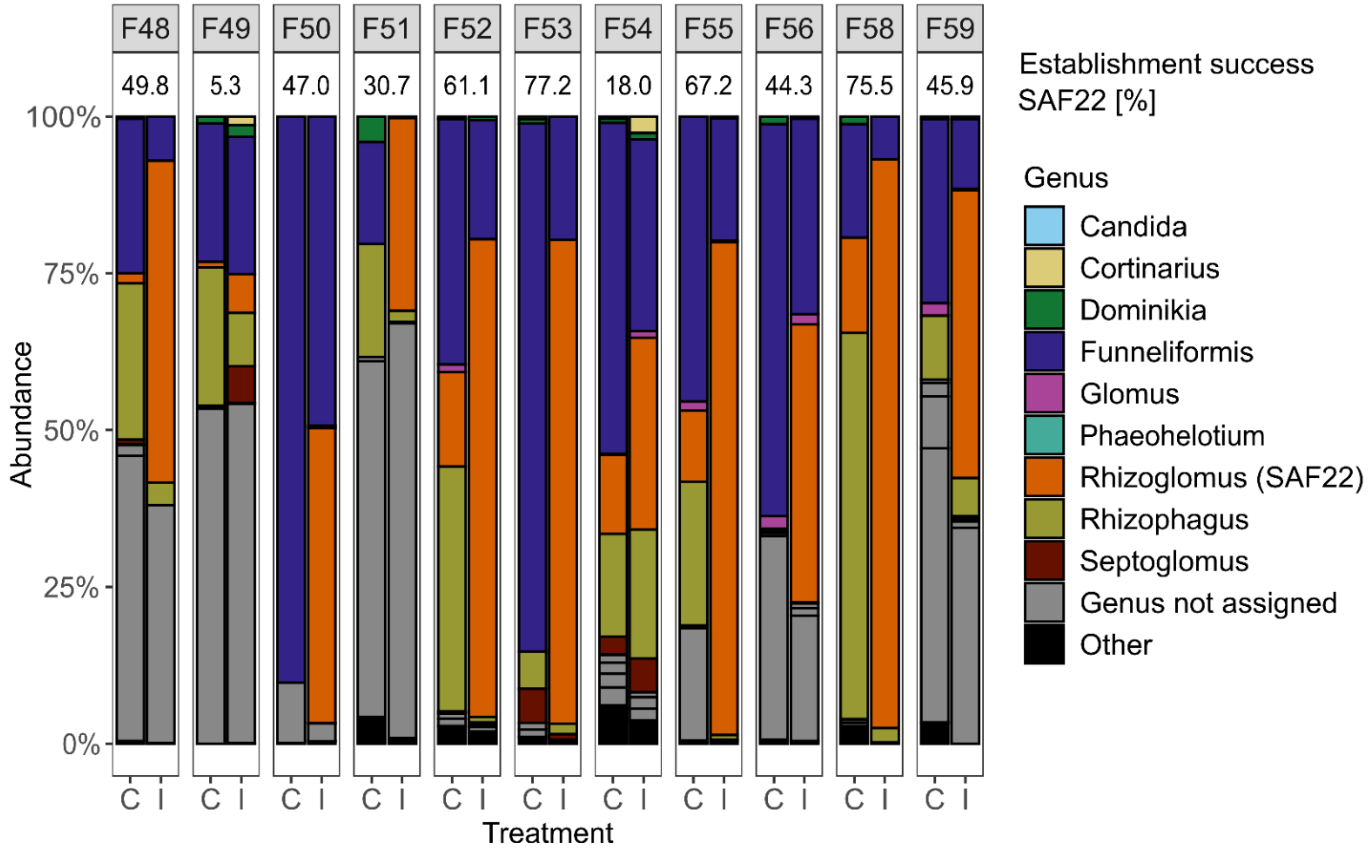

Supplementary Fig. 4: Relationship between all pathogen sOTUs and MGR.

Fungal guilds of all sOTUs were assessed and the abundance of pathogenic OTUs were summed up. The scatter plot on the left refers only to the guild "plant pathogen", while the plot on the right summarises all guilds with the keyword "plant pathogen". No significant correlations were found. On the one hand, this could be because in most cases there are no exact matches in the sequence databases and because even within a species, traits are often highly diverse. Therefore, functional annotations are often unreliable if not conducted on the strain level. On the other hand, it may indeed be that only a few pathogens are important in the studied context of one particular AMF strain and maize as host plant. AMF strains are likely specialised in their ability to protect against specific pathogens. The correlation coefficients (Spearman rank) and the significant values (p) are displayed in the plots. The blue shaded area represents the 95% confidence interval.

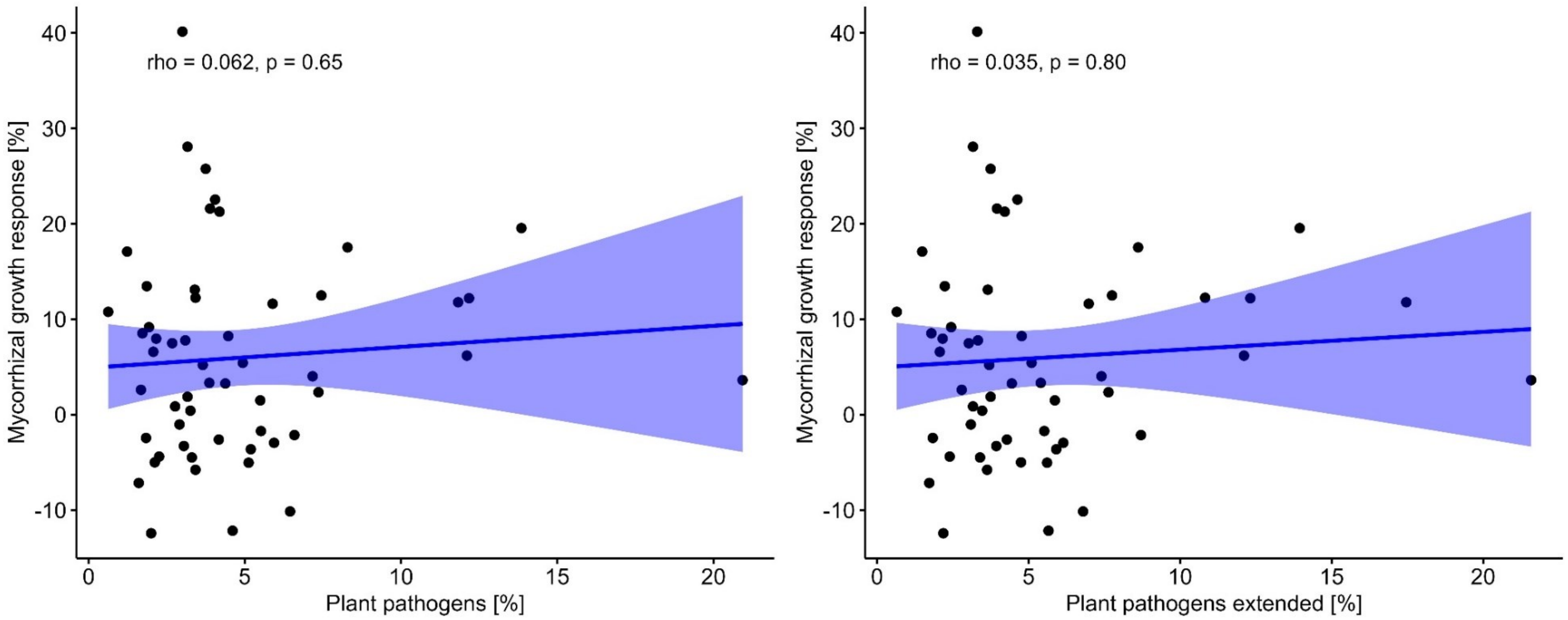

Supplementary Fig. 5: Correlation of plant biomass and yield data.

The grey shaded area represents the 95% confidence interval. The correlation coefficient (Spearman rank, rho) and the significant value (p) is displayed in the plot.

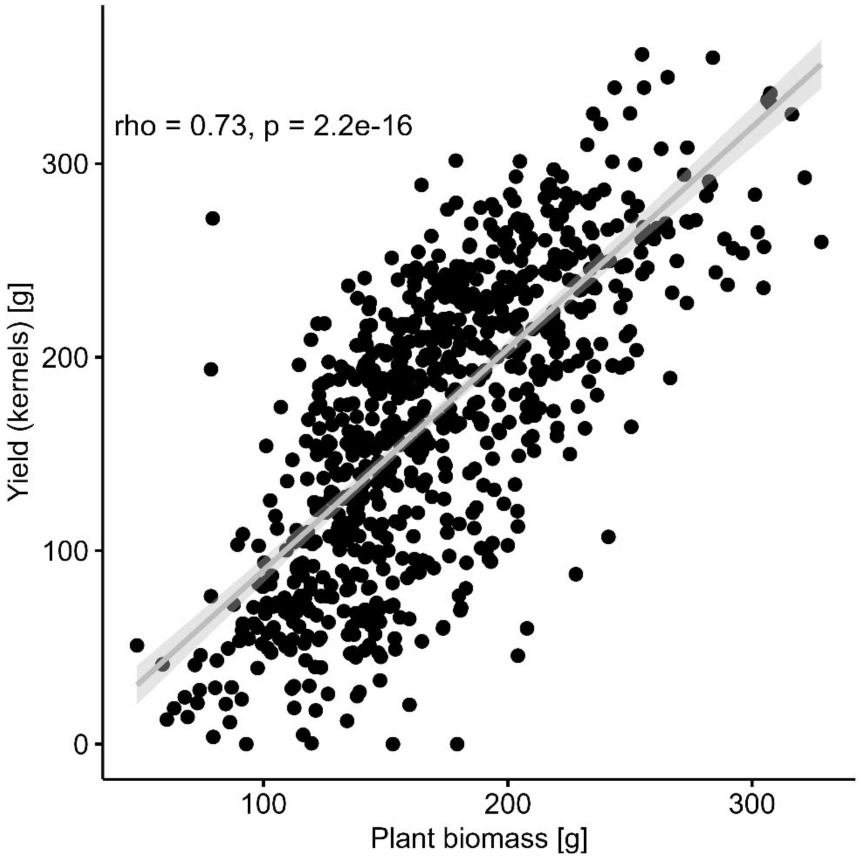

Supplementary Table 1: Overview of inoculated fields including corresponding mycorrhizal growth response and soil parameters.

See separate spreadsheet "Supplementary Tables".

Supplementary Table 2: Pairwise correlations of soil parameters and MGR.

Table show Spearman rank correlations coefficients and associated p-values in increasing order. No significant ($p<0.05$) correlations were found. Magnesium, Corg, humus and Ntot were weakly correlated with MGR.

	r	p
**magnesium_H2O_lbu**	0.260	0.057
**Corg_agro**	-0.258	0.060
**humus_agro**	-0.252	0.066
**Ntot_agro**	-0.262	0.089
**Ctot_agro**	-0.242	0.118
**clay_lbu**	-0.214	0.120
**silt_lbu**	-0.198	0.150
**clay_agro**	-0.182	0.187
**WHC_agro**	-0.172	0.215
**sand_agro**	0.156	0.259
**Nmin_agro**	0.154	0.265
**ammonium_H2O_lbu**	-0.173	0.268
**calcium_agro**	-0.152	0.271
**respiration_lbu**	-0.153	0.273
**nitrate_agro**	0.148	0.284
**slan_lbu**	-0.147	0.292
**CEC_agro**	-0.142	0.307
**calcium_EDTA_lbu**	-0.154	0.325
**iron_H2O_lbu**	0.153	0.327
**Ptot_agro**	-0.135	0.332
**respiration_agro**	-0.131	0.343
**iron_EDTA_lbu**	0.131	0.345
**sodium_agro**	0.122	0.379
**fertility_lbu**	-0.123	0.390
**cMIC_agro**	-0.118	0.394
**nitrate_H2O_lbu**	0.129	0.411
**potassium_EDTA_lbu**	-0.112	0.419
**magnesium_CC_lbu**	0.108	0.439
**nMIC_agro**	-0.096	0.491
**ph_lbu**	0.093	0.502
**ammonium_agro**	0.075	0.591
**P_olsen_lbu**	-0.082	0.605
**hydrogen_agro**	-0.070	0.613
**sodium_H2O_lbu**	0.078	0.621
**magnesium_agro**	0.055	0.694
**phosphorus_CO2_lbu**	-0.051	0.717
**copper_EDTA_lbu**	0.045	0.746
**boron_EDTA_lbu**	-0.041	0.771
**silt_agro**	-0.037	0.791
**magnesium_EDTA_lbu**	-0.036	0.794
**potassium_agro**	-0.027	0.845

**manganese_EDTA_lbu**	-0.027	0.847
**boron_H2O_lbu**	-0.027	0.865
**phosphorus_EDTA_lbu**	-0.020	0.888
**phosphorus_H2O_lbu**	-0.013	0.925
**potassium_CO2_lbu**	0.013	0.926
**vast_lbu**	-0.012	0.932
**zinc_EDTA_lbu**	-0.008	0.959
**humus_lbu**	-0.006	0.964
**calcium_H2O_lbu**	0.006	0.967
**BS_agro**	-0.005	0.972
**potassium_H2O_lbu**	0.004	0.978

Supplementary Table 3: Overview of co-correlated parameters.

The 22 parameters on the left were kept for further model selection, whereas parameters on the right were filtered out since they were strongly co-correlated (r>0.8 or r<-0.8).

Selected parameters	Co-correlated parameters and their respective correlation coefficients
**Ptot_agro**	
**potassium_agro**	potassium_CO2_lbu (0.88), potassium_H2O_lbu (0.91), potassium_EDTA_lbu (0.93)
**pH_lbu**	BS_agro (0.88), hydrogen_agro (-0.86)
**Corg_agro**	CEC_agro (0.80), WHC_agro (0.80), humus_agro (1.0)
**humus_lbu**	
**sodium_agro**	
**magnesium_H20_lbu**	magnesium_agro (0.85), magnesium_CC_lbu (0.83)
**magnesium_EDTA_lbu**	
**calcium_agro**	calcium_H2O_lbu (0.83), CEC_agro (0.87), respiration_agro (0.82)
**phosphorus_H2O_lbu**	phosphorus_CO2_lbu (0.93), phosphorus_EDTA_lbu (0.86)
**manganese_EDTA_lbu**	
**boron_EDTA_lbu**	
**copper_EDTA_lbu**	
**iron_EDTA_lbu**	
**cMIC_agro**	nMIC_agro (0.91), respiration_agro (0.83)
**Nmin_agro**	nitrate_agro (0.99)
**ammonium_agro**	
**sand_agro**	
**silt_agro**	
**clay_agro**	
**silt_lbu**	
**clay_lbu**	

Supplementary Table 4: Random forest analysis of soil parameters.

The analysis was conducted on the reduced pool of 22 parameters (see Supplementary Table 3). Parameters are sorted by descending importance (IncNodePurity).

Soil parameter	IncNodePurity
**sand_agro**	488
**silt_agro**	467
**magnesium_H2O_lbu**	420
**magnesium_EDTA_lbu**	348
**Ptot_agro**	324
**cMIC_agro**	313
**Corg_agro**	311
**iron_EDTA_lbu**	306
**manganese_EDTA_lbu**	303
**Nmin_agro**	256
**clay_agro**	213
**boron_EDTA_lbu**	197
**copper_EDTA_lbu**	192
**phosphorus_H2O_lbu**	186
**ph_lbu**	184
**potassium_agro**	181
**humus_lbu**	179
**ammonium_agro**	154
**calcium_agro**	147
**sodium_agro**	140
**silt_lbu**	103
**clay_lbu**	90

Supplementary Table 5: Soil parameters selected for final model input.

Comparison of the most important predictors of MGR identified by the glmulti, stepAIC and random forest analyses. The selection by the respective methods is shown with a grey background. The *glmulti* analysis was limited to a maximum number of ten predictors. Similarly, for the random forest analysis, only the top ten predictors are shown.

**Soil parameter**	**Parameter name in main text**	**glmulti (top 10)**	**stepAIC**	**Random forest (top 10)**
magnesium_EDTA_lbu	magnesium(EDTA)			
magnesium_H2O_lbu	magnesium(H2O)			
manganese_EDTA_lbu	Manganese			
Nmin_agro	Nmin			
iron_EDTA_lbu	Iron			
cMIC_agro	cMIC			
phosphorus_H2O_lbu	phosphorus(H2O)			
ammonium_agro	Ammonium			
Corg_agro	Corg			
sand_agro	Sand			
silt_agro	Silt			
Ptot_agro	Ptot			
boron_EDTA_lbu	Boron			
clay_lbu	Clay			
pH_lbu	pH			

Supplementary Table 6: Soil OTU table.

Table shows merged OTU abundances for the four replicates of each samples and rarefied to an even sampling depth (i.e., 4272 reads). The fungal community was characterized by a high relative abundance of Ascomycota, followed by Mortierellomycota and Basidiomycota. The relative abundance of Glomeromycota was very low and ranged from 0 to 1.8%. See separate spreadsheet “Supplementary Tables”.

Supplementary Table 7: Results of the indicator species analysis of fields with low and high MGR.

Displayed are soil OTUs that were associated with low or high MGR (Spearman rank correlation, p<0.1).

OTU	MGR	statistic	p-value	Taxonomy
**OTU100**	High	0.327	0.086	Ascomycota; Pezizomycetes; Pezizales; Pyronemataceae; Byssonectria; Byssonectria_fusispora
**OTU16**	High	0.341	0.058	Ascomycota; Sordariomycetes; Hypocreales; Nectriaceae; Gibberella; Gibberella_intricans
**OTU177**	High	0.249	0.047	Ascomycota; Sordariomycetes; Sordariales; Chaetomiaceae; Humicola; Humicola_nigrescens
**OTU18**	High	0.305	0.089	Basidiomycota; Tremellomycetes;
**OTU243**	High	0.349	0.096	NA
**OTU274**	High	0.255	0.037	Ascomycota; Saccharomycetes; Saccharomycetales; Phaffomycetaceae; Cyberlindnera; Cyberlindnera_misumaiensis
**OTU38**	High	0.333	0.084	Basidiomycota; Tremellomycetes; Cystofilobasidiales; Mrakiaceae; Mrakia; Mrakia_frigida
**OTU388**	High	0.328	0.095	Ascomycota; Sordariomycetes; Hypocreales; Stachybotryaceae; Striaticonidium; Striaticonidium_humicola
**OTU49**	High	0.365	0.081	Olpidiomycota; Olpidiomycetes; Olpidiales; Olpidiaceae; Olpidium; Olpidium_brassicae
**OTU561**	High	0.358	0.093	NA
**OTU106**	Low	0.277	0.080	NA
**OTU142**	Low	0.374	0.048	Ascomycota; Leotiomycetes; Helotiales; Helotiaceae; Phaeohelotium; NA
**OTU15**	Low	0.311	0.097	Ascomycota
**OTU179**	Low	0.358	0.069	Ascomycota; Leotiomycetes; Helotiales
**OTU182**	Low	0.329	0.099	Ascomycota; Dothideomycetes; Pleosporales; Torulaceae; Dendryphion; Dendryphion_europaeum
**OTU217**	Low	0.523	0.008	NA
**OTU227**	Low	0.335	0.092	Ascomycota; Leotiomycetes; Helotiales; Helotiaceae; Phaeohelotium
**OTU273**	Low	0.353	0.080	Ascomycota; Eurotiomycetes; Chaetothyriales; Herpotrichiellaceae
**OTU366**	Low	0.448	0.039	NA
**OTU392**	Low	0.388	0.094	Chytridiomycota; Spizellomycetes; Spizellomycetales; Powellomycetaceae; Powellomyces
**OTU451**	Low	0.347	0.100	Ascomycota; Sordariomycetes; Hypocreales; Hypocreales_fam_Incertae_sedis; Acremonium; Acremonium_tubakii
**OTU544**	Low	0.313	0.099	Ascomycota; Leotiomycetes; Helotiales; Helotiaceae; Phaeohelotium
**OTU58**	Low	0.348	0.092	NA

**OTU589**	Low	0.376	0.039	NA
**OTU608**	Low	0.408	0.04	Ascomycota; Sordariomycetes; Sordariales; Chaetomiaceae; Dichotomopilus; Dichotomopilus_erectus
**OTU68**	Low	0.407	0.038	Ascomycota; Dothideomycetes; Pleosporales; Phaeosphaeriaceae
**OTU69**	Low	0.378	0.048	Ascomycota; Leotiomycetes; Helotiales; Helotiaceae; Phaeohelotium
**OTU72**	Low	0.434	0.030	Basidiomycota; Agaricomycetes; Agaricales; Psathyrellaceae; Psathyrella; Psathyrella_sacchariolens
**OTU884**	Low	0.339	0.084	Ascomycota; Eurotiomycetes; Onygenales

Supplementary Table 8: Results of the DESeq2 analysis of soil OTUs.

Table shows soil OTUs with differential abundance between low and high MGR fields (Wald test, adjusted for multiple comparison, p<0.1). A negative log2FC corresponds to a higher relative abundance in high MGR fields, whereas a positive log2FC corresponds to a higher relative abundance in low MGR fields.

OTU ID	baseMean	log2FC	lfcSE	stat	pvalue	padj	Taxonomy
**OTU156**	5.551	-2.740	0.738	-3.712	0.0001	0.083	Chytridiomycota, Rhizophlyctidomycetes, Rhizophlyctidales, Rhizophlyctidaceae
**OTU18**	56.013	-2.597	0.762	-3.410	0.001	0.083	Basidiomycota, Tremellomycetes
**OTU251**	3.269	-2.402	0.722	-3.326	0.0001	0.048	Chytridiomycota, Cladochytriomycetes, Cladochytriales, Cladochytriaceae, Cladochytrium
**OTU100**	4.154	-2.252	0.665	-3.388	0.001	0.083	Ascomycota, Pezizomycetes, Pezizales, Pyronemataceae, Byssonectria_fusispora
**OTU253**	2.614	-2.107	0.719	-2.930	0.003	0.095	Ascomycota, Eurotiomycetes, Onygenales, Onygenales_fam_Incertae_sedis, Chrysosporium_lobatum
**OTU242**	2.185	1.716	0.588	2.917	0.004	0.095	Ascomycota, Sordariomycetes, Hypocreales, Hypocreales_fam_Incertae_sedis, Sarocladium_strictum
**OTU273**	2.400	1.772	0.574	3.087	0.002	0.083	Ascomycota, Eurotiomycetes, Chaetothyriales, Herpotrichiellaceae
**OTU281**	2.364	1.865	0.612	3.046	0.002	0.083	Ascomycota, Sordariomycetes, Hypocreales, Bionectriaceae, Clonostachys_phyllophila
**OTU74**	3.581	1.926	0.647	2.976	0.003	0.090	NA
**OTU252**	3.116	1.939	0.639	3.033	0.002	0.083	Basidiomycota, Agaricomycetes, Trechisporales, Hydnodontaceae
**OTU631**	2.471	1.948	0.617	3.154	0.002	0.083	NA
**OTU134**	2.579	2.026	0.669	3.026	0.002	0.083	Ascomycota, Leotiomycetes, Helotiales, Helotiaceae
**OTU268**	2.901	2.122	0.679	3.126	0.002	0.083	Ascomycota, Eurotiomycetes, Onygenales
**OTU375**	2.794	2.170	0.657	3.302	0.001	0.083	NA
**OTU80**	9.025	2.224	0.718	3.100	0.002	0.083	Basidiomycota, Tremellomycetes, Cystofilobasidiales, Cystofilobasidiales_fam_Incertae_sedis, Mrakiella_aquatica
**OTU75**	3.510	2.252	0.709	3.175	0.001	0.083	NA

Supplementary Table 9: Random forest analysis of soil OTUs.

The most important soil OTUs (IncNodePurity > 30) for the prediction of MGR are displayed in descending order.

OTU ID	IncNodePurity
**OTU151**	359
**OTU18**	285
**OTU26**	202
**OTU15**	177
**OTU1325**	122
**OTU724**	109
**OTU181**	91
**OTU52**	89
**OTU529**	82
**OTU38**	75
**OTU9**	75
**OTU194**	67
**OTU147**	65
**OTU182**	64
**OTU24**	57
**OTU39**	53
**OTU322**	46
**OTU728**	41
**OTU31**	40
**OTU422**	40
**OTU318**	39
**OTU3**	38
**OTU30**	36
**OTU58**	35
**OTU32**	35
**OTU33**	34
**OTU25**	34
**OTU16**	32
**OTU34**	31
**OTU211**	31
**OTU110**	31
**OTU40**	31
**OTU273**	31
**OTU41**	30
**OTU155**	30

Supplementary Table 10: Soil fungal OTUs selected for final model input.

Comparison of the 44 soil OTUs selected (grey background) by the indicator species (31 sOTUs), DESeq2 (19 sOTUs) and random forest analyses (continuous ranking of sOTUs). Within the top 30 sOTUS identified by random forest, only 5 sOTUs were overlapping with the indicator species and DESeq2 analyses. The combined results of the indicator species and DESeq2 analyses (44 sOTUs, no further sOTUs of the random forest analysis where added in order to not inflate the final pool of sOTUs) were subjected to a further selection step with glmulti. The resulting final 13 sOTUs are highlighted in bold. In addition to the automated taxonomic assignments, these sequences were searched against the NCBI BLAST database to obtain a more refined taxonomic identification.

MGR	OTU ID	Indicator species	DESeq2	Random forest	Taxonomy	NCBI BLAST (% identity)
High	**sOTU16**				Ascomycota; Sordariomycetes; Hypocreales; Nectriaceae; Gibberella; Gibberella_intricans	Fusarium equiseti (100%)
	**sOTU18**				Basidiomycota; Tremellomycetes	Trichosporon sp. (100%)
	sOTU38				Basidiomycota; Tremellomycetes; Cystofilobasidiales; Mrakiaceae; Mrakia; Mrakia_frigida	
	**sOTU49**				Olpidiomycota; Olpidiomycetes; Olpidiales; Olpidiaceae; Olpidium; Olpidium_brassicae	Olpidium brassicae (99.83%)
	sOTU93				Ascomycota; Sordariomycetes; Glomerellales; Plectosphaerellaceae; Plectosphaerella	
	sOTU100				Ascomycota; Pezizomycetes; Pezizales; Pyronemataceae; Byssonectria; Byssonectria_fusispora	
	sOTU156				Chytridiomycota, Rhizophlyctidomycetes, Rhizophlyctidales, Rhizophlyctidaceae	
	**sOTU177**				Ascomycota; Sordariomycetes; Sordariales; Chaetomiaceae; Humicola; Humicola_nigrescens	*Chaetomium* sp. (98.83%)
	sOTU243				NA	
	**sOTU251**				Chytridiomycota; Cladochytriomycetes; Cladochytriales; Cladochytriaceae; Cladochytrium	No blast hit
	sOTU253				Ascomycota; Eurotiomycetes; Onygenales; Onygenales_fam_Incertae_sedis; Chrysosporium; Chrysosporium_lobatum	
	sOTU274				Ascomycota; Saccharomycetes; Saccharomycetales; Phaffomycetaceae; Cyberlindnera; Cyberlindnera_misumaiensis	
	**sOTU388**				Ascomycota; Sordariomycetes; Hypocreales; Stachybotryaceae; Striaticonidium; Striaticonidium_humicola	Striaticonidium sp./ Myrothecium sp. (99.64%)

	**sOTU561**				NA	No blast hit
Low	OTU15				Ascomycota	
	**OTU58**				NA	No blast hit
	**OTU68**				Ascomycota; Dothideomycetes; Pleosporales; Phaeosphaeriaceae	Phaeosphaeria sp./Dokmaia sp. (100%)
	OTU69				Ascomycota; Leotiomycetes; Helotiales; Helotiaceae; Phaeohelotium	
	OTU72				Basidiomycota; Agaricomycetes; Agaricales; Psathyrellaceae; Psathyrella; Psathyrella_sacchariolens	
	OTU106				NA	
	OTU134				Ascomycota; Leotiomycetes; Helotiales; Helotiaceae; Phaeohelotium	
	**OTU142**				Ascomycota; Leotiomycetes; Helotiales; Helotiaceae; Phaeohelotium	No blast hit
	OTU179				Ascomycota; Leotiomycetes; Helotiales; Helotiaceae; Phaeohelotium	
	OTU182				Ascomycota; Dothideomycetes; Pleosporales; Torulaceae; Dendryphion; Dendryphion_europaeum	
	OTU217				NA	
	**OTU227**				Ascomycota; Leotiomycetes; Helotiales; Helotiaceae; Phaeohelotium	No blast hit
	OTU243				Ascomycota, Sordariomycetes, Hypocreales, Hypocreales_fam_Incertae_sedis, Sarocladium_strictum	
	OTU252				Basidiomycota; Agaricomycetes; Trechisporales; Hydnodontaceae; Trechispora	
	OTU268				Ascomycota, Eurotiomycetes, Onygenales	
	**OTU273**				Ascomycota; Eurotiomycetes; Chaetothyriales; Herpotrichiellaceae	
	OTU281				Ascomycota, Sordariomycetes, Hypocreales, Bionectriaceae, Clonostachys_phyllophila	
	OTU284				Basidiomycota; Agaricomycetes; Boletales; Boletaceae; Indoporus; Indoporus shoreae	
	OTU366				NA	
	OTU375				NA	
	**OTU392**				Chytridiomycota; Spizellomycetes; Spizellomycetales; Powellomycetaceae; Powellomyces	No blast hit
	OTU451				Ascomycota; Sordariomycetes; Hypocreales; Hypocreales_fam_Incertae_sedis; Acremonium; Acremonium_tubakii	
	OTU544				Ascomycota; Leotiomycetes; Helotiales; Helotiaceae; Phaeohelotium	

OTU589			NA
OTU608			Ascomycota; Sordariomycetes; Sordariales; Chaetomiaceae; Dichotomopilus; Dichotomopilus_erectus
OTU631			NA
OTU74			NA
OTU75			NA
OTU80			Basidiomycota, Tremellomycetes, Cystofilobasidiales, Cystofilobasidiales_fam_Incertae_sedis, Mrakiella_aquatica
OTU884			Ascomycota; Eurotiomycetes; Onygenales

Supplementary Table 11: Root OTU table.

Table shows OTU abundances rarefied to an even sampling depth (i.e., 1660 reads). See separate spreadsheet “Supplementary Tables”.

Supplementary Table 12: AMF establishment success.

On the left, the summed relative abundance of all root OTUs corresponding to SAF22 (rOTU2, rOTU4, rOTU9, rOTU16, rOTU74, rOTU84, rOTU165) is shown for the control (CTL) and inoculated (AMF) samples, as well as their difference (Δ). On the right, the averaged (eight replicates) total root colonisation of the control and inoculated samples is shown, as well as their difference (Δ).

Field	Relative abundance of SAF22 rOTUS			Total root colonisation		
	CTL [%]	AMF [%]	Δ (AMF - CTL) [%]	CTL_mean [%]	AMF_mean [%]	Δ (AMF-CTL) [%]
**F01**	13.0	60.8	47.9	66.8	81.9	15.1
**F04**	0.1	59.9	59.8	70.9	83.3	12.4
**F05**	0.0	0.7	0.7	76.3	86.0	9.7
**F06**	0.7	0.0	0.0	61.9	56.1	-5.7
**F07**	0.0	9.4	9.4	56.6	70.5	13.9
**F08**	0.0	48.8	48.8	45.1	72.6	27.5
**F09**	0.0	8.1	8.1	61.9	70.6	8.7
**F10**	0.9	0.4	0.0	57.5	54.0	-3.5
**F12**	0.0	0.0	0.0	81.6	92.3	10.6
**F13**	0.0	64.8	64.8	38.1	68.9	30.8
**F14**	0.0	23.7	23.7	81.9	75.3	-6.6
**F16**	14.5	61.9	47.4	82.4	80.3	-2.1
**F17**	0.0	81.6	81.6	62.1	87.3	25.1
**F18**	0.0	99.2	99.2	27.5	96.8	69.3
**F19**	0.0	2.0	2.0	45.9	59.9	14.0
**F20**	0.0	97.7	97.7	21.1	51.0	29.9
**F21**	3.0	20.8	17.9	74.4	75.4	1.1
**F22**	0.0	0.0	0.0	53.3	56.0	2.8
**F23**	0.0	36.3	36.3	59.8	80.1	20.4
**F24**	0.0	0.0	0.0	24.1	43.1	19.0
**F25**	0.0	76.4	76.4	41.0	87.0	46.0
**F26**	0.0	0.0	0.0	44.6	40.1	-4.4
**F27**	0.0	75.4	75.4	73.4	91.4	18.0
**F28**	0.0	0.0	0.0	51.8	66.1	14.4
**F29**	0.0	65.8	65.8	52.0	80.6	28.6
**F30**	5.4	50.3	44.9	68.6	80.0	11.4
**F31**	0.0	17.8	17.8	64.1	90.8	26.6
**F32**	0.0	56.6	56.6	56.8	83.5	26.8
**F33**	0.0	52.4	52.4	67.1	77.9	10.8
**F34**	0.0	0.0	0.0	70.0	64.6	-5.4
**F35**	0.0	45.0	45.0	55.1	80.5	25.4
**F36**	0.0	0.0	0.0	55.5	53.9	-1.6
**F37**	0.0	28.6	28.6	47.3	73.9	26.6
**F38**	0.0	97.3	97.3	82.9	96.6	13.8

**F39**	0.0	82.2	82.2	56.3	79.6	23.3
**F40**	17.5	81.5	64.0	68.5	88.8	20.3
**F41**	0.0	0.0	0.0	62.6	67.0	4.4
**F42**	0.0	0.0	0.0	40.9	50.3	9.4
**F43**	0.0	27.4	27.4	34.6	55.5	20.9
**F44**	43.1	80.1	37.0	63.4	90.6	27.2
**F45**	0.0	99.1	99.1	74.4	89.3	14.8
**F46**	0.0	63.3	63.3	68.6	87.6	18.9
**F47**	0.0	60.5	60.5	54.3	57.8	3.5
**F48**	1.6	51.3	49.8	39.6	70.3	30.7
**F49**	0.9	6.2	5.3	76.4	69.3	-7.1
**F50**	0.0	47.0	47.0	74.8	89.4	14.7
**F51**	0.0	30.7	30.7	21.0	57.0	36.0
**F52**	15.1	76.1	61.1	84.5	92.5	8.0
**F53**	0.0	77.2	77.2	59.8	89.5	29.8
**F54**	12.7	30.6	18.0	95.2	87.0	-8.2
**F55**	11.4	78.6	67.2	78.2	96.0	17.8
**F56**	0.0	44.3	44.3	73.8	72.9	-0.9
**F58**	15.2	90.7	75.5	44.6	88.3	43.6
**F59**	0.0	45.9	45.9	78.0	79.5	1.5

Supplementary Table 13: Fungal guilds of soil OTUs.

See separate spreadsheet “Supplementary Tables”.

Supplementary Table 14: Results of the DESeq2 analysis of root OTUs in low MGR fields.

Table shows root OTUs with a significant (Wald test, adjusted for multiple comparison, $p < 0.05$) differential abundance between control and inoculated samples. A negative log2FC corresponds to a higher relative abundance in control samples, whereas a positive log2FC corresponds to a higher relative abundance in inoculated samples. In addition, the values for rOTU20, which likely matches the important soil predictor sOTU18, are highlighted in grey.

OTU ID	baseMean	log2FC	lfcSE	Stat	Pvalue	Padj	Taxonomy
**OTU8**	4.727	-2.168	0.369	-5.883	0.0001	0.0001	Glomeromycetes, Glomerales, Glomeraceae, Rhizophagus, Rhizophagus irregularis
**OTU5**	7.582	-1.999	0.363	-5.505	0.0001	0.0001	Glomeromycetes, Glomerales, Glomeraceae, Funneliformis, Funneliformis sp.
**OTU10**	3.780	-1.484	0.344	-4.312	0.0001	0.0001	Glomeromycetes, Glomerales, Glomeraceae, Rhizophagus, Rhizophagus sp.
**OTU13**	2.202	-1.348	0.307	-4.394	0.0001	0.0001	Glomeromycetes, Glomerales, Glomeraceae, Rhizophagus, Rhizophagus irregularis
**OTU19**	2.329	-1.284	0.316	-4.066	0.0001	0.0001	Glomeromycetes, Glomerales, Glomeraceae, Rhizophagus, Rhizophagus irregularis
**OTU21**	1.567	-0.973	0.316	-3.075	0.002	0.013	Glomeromycetes
**OTU3**	18.010	-0.970	0.392	-2.479	0.013	0.059	Glomeromycetes, Glomerales, Glomeraceae, Funneliformis, Funneliformis mosseae
**OTU38**	1.568	-0.895	0.283	-3.161	0.002	0.011	Glomeromycetes, Glomerales, Glomeraceae, Rhizophagus, Rhizophagus irregularis
**OTU49**	1.459	-0.811	0.300	-2.705	0.007	0.036	Glomeromycetes, Glomerales, Glomeraceae, Rhizophagus, Rhizophagus sp.
**OTU28**	1.451	-0.755	0.285	-2.649	0.008	0.039	Glomeromycetes, Glomerales, Glomeraceae, Funneliformis, Funneliformis sp.
**OTU34**	1.599	-0.674	0.294	-2.291	0.022	0.090	Glomeromycetes, Glomerales, Glomeraceae, Glomus, Glomus sp.
**OTU98**	1.342	-0.656	0.294	-2.231	0.026	0.090	Sordariomycetes, Glomerellales, Plectosphaerellaceae, Verticillium
**OTU43**	1.411	-0.607	0.282	-2.153	0.031	0.099	Tremellomycetes
**OTU25**	1.313	-0.603	0.270	-2.235	0.025	0.090	Paraglomeromycetes, Paraglomerales, Paraglomeraceae, Paraglomus
**OTU20**	1.197	0.109	0.263	0.415	0.678	0.837	100% similarity with full length of soil OTU18 (Trichosporon sp.)
**OTU50**	1.342	0.628	0.292	2.153	0.031	0.099	Glomeromycetes, Glomerales, Glomeraceae, Dominikia, Dominikia iranica
**OTU29**	1.518	0.660	0.291	2.266	0.023	0.090	Glomeromycetes, Glomerales, Glomeraceae, Septoglomus, Septoglomus viscosum

**OTU4**	9.592	1.098	0.383	2.869	0.004	0.024	Glomeromycetes,	Glomerales,	Glomeraceae,	Rhizoglomus,	Rhizoglomus irregulare SAF22
**OTU16**	2.558	1.925	0.287	6.696	0.0001	00.001	Glomeromycetes,	Glomerales,	Glomeraceae,	Rhizoglomus,	Rhizoglomus irregulare SAF22
**OTU9**	4.254	2.764	0.332	8.317	0.0001	0.0001	Glomeromycetes,	Glomerales,	Glomeraceae,	Rhizoglomus,	Rhizoglomus irregulare SAF22
**OTU2**	25.142	5.302	0.345	15.363	0.0001	0.0001	Glomeromycetes,	Glomerales,	Glomeraceae,	Rhizoglomus,	Rhizoglomus irregulare SAF22

Supplementary Table 15: Results of the DESeq2 analysis of root OTUs in high MGR fields.

Table shows root OTUs with a significant (Wald test, adjusted for multiple comparison, p <0.05) differential abundance between control and inoculated samples. A negative log2FC corresponds to a higher relative abundance in control samples, whereas a positive log2FC corresponds to a higher relative abundance in inoculated samples. In addition, the values for OTU20, which likely matches the important soil predictor OTU18, are highlighted in grey.

OTU ID	baseMean	log2FC	lfcSE	Stat	pvalue	Padj	Taxonomy
**OTU19**	17.890	-3.163	0.465	-6.804	0	0	Glomeromycetes, Glomerales, Glomeraceae, Rhizophagus, Rhizophagus irregularis
**OTU26**	8.096	-3.052	0.415	-7.355	0	0	Glomeromycetes, Glomerales, Glomeraceae, Rhizophagus, Rhizophagus sp.
**OTU13**	10.887	-2.801	0.432	-6.488	0	0	Glomeromycetes, Glomerales, Glomeraceae, Rhizophagus, Rhizophagus irregularis
**OTU38**	5.362	-2.729	0.404	-6.759	0	0	Glomeromycetes, Glomerales, Glomeraceae, Rhizophagus, Rhizophagus irregularis
**OTU8**	37.470	-2.561	0.528	-4.855	0	0	Glomeromycetes, Glomerales, Glomeraceae, Rhizophagus, Rhizophagus irregularis
**OTU5**	64.335	-2.455	0.509	-4.825	0	0	Glomeromycetes, Glomerales, Glomeraceae, Funneliformis, Funneliformis sp.
**OTU10**	27.822	-2.022	0.506	-4.000	0	0	Glomeromycetes, Glomerales, Glomeraceae, Rhizophagus, Rhizophagus sp.
**OTU17**	4.008	-1.829	0.390	-4.687	0	0	Glomeromycetes, Glomerales, Claroideoglomeraceae
**OTU24**	3.506	-1.617	0.385	-4.195	0	0	Glomeromycetes, Glomerales, Glomeraceae, Rhizoglomus, Rhizoglomus silesianum
**OTU51**	2.380	-1.590	0.357	-4.451	0	0	Glomeromycetes, Glomerales, Glomeraceae, Rhizophagus, Rhizophagus irregularis
**OTU28**	2.476	-1.543	0.343	-4.505	0	0	Glomeromycetes, Glomerales, Glomeraceae, Funneliformis, Funneliformis sp.
**OTU63**	2.206	-1.405	0.329	-4.275	0	0	Glomeromycetes, Glomerales, Glomeraceae, Rhizophagus, Rhizophagus irregularis
**OTU3**	172.515	-1.197	0.515	-2.323	0.020	0.062	Glomeromycetes, Glomerales, Glomeraceae, Funneliformis, Funneliformis mosseae
**OTU34**	3.946	-1.176	0.387	-3.037	0.002	0.011	Glomeromycetes, Glomerales, Glomeraceae, Glomus, Glomus sp.
**OTU11**	11.000	-1.088	0.494	-2.204	0.027	0.073	Glomeromycetes, Glomerales, Glomeraceae, Funneliformis, Funneliformis caledonium
**OTU83**	2.452	-1.072	0.326	-3.288	0.001	0.005	Leotiomycetes, Helotiales, Helotiaceae, Tetracladium, Tetracladium sp.
**OTU25**	2.593	-1.017	0.361	-2.818	0.005	0.019	Paraglomeromycetes, Paraglomerales, Paraglomeraceae, Paraglomus
**OTU104**	1.677	-0.926	0.302	-3.067	0.002	0.01	Glomeromycetes, Glomerales, Glomeraceae, Funneliformis, Funneliformis sp.
**OTU58**	2.257	-0.919	0.310	-2.960	0.003	0.013	Dothideomycetes, Capnodiales, Cladosporiaceae, Cladosporium, Cladosporium spp.
**OTU132**	1.541	-0.847	0.291	-2.909	0.004	0.015	Tremellomycetes, Tremellales, Bulleribasidiaceae, Vishniacozyma, Vishniacozyma sp.
**OTU98**	1.721	-0.764	0.304	-2.514	0.012	0.039	Sordariomycetes, Glomerellales, Plectosphaerellaceae, Verticillium
**OTU68**	1.769	-0.760	0.292	-2.607	0.009	0.032	Dothideomycetes, Pleosporales, Cucurbitariaceae, Pyrenochaeta
**OTU69**	2.529	-0.726	0.322	-2.255	0.024	0.069	Glomeromycetes, Glomerales, Glomeraceae, Rhizophagus, Rhizophagus irregularis
**OTU111**	1.510	-0.721	0.287	-2.512	0.012	0.039	Leotiomycetes, Helotiales, Helotiales fam Incertae sedis, Mycochaetophora, Mycochaetophora gentianae

**OTU81**	2.681	-0.717	0.336	-2.134	0.033	0.082	Olpidiomycetes, Olpidiales, Olpidiaceae, Olpidium, Olpidium brassicae
**OTU145**	1.419	-0.656	0.272	-2.411	0.016	0.050	Leotiomycetes, Helotiales, Helotiaceae, Phaeohelotium
**OTU133**	1.392	-0.649	0.298	-2.178	0.029	0.076	NA
**OTU150**	1.328	-0.628	0.282	-2.225	0.026	0.073	Agaricomycetes, Agaricales, Psathyrellaceae, Coprinopsis, Coprinopsis vermiculifer
**OTU144**	1.419	-0.572	0.279	-2.053	0.040	0.095	Glomeromycetes, Glomerales, Claroideoglomeraceae, Claroideoglomus
**OTU116**	1.290	-0.554	0.275	-2.015	0.044	0.099	NA
**OTU20**	2.401	-0.640	0.338	-1.894	0.058	0.129	100% similarity with full length of soil OTU18 (Trichosporon sp.)
**OTU134**	1.399	0.591	0.286	2.066	0.039	0.095	NA
**OTU50**	1.547	0.622	0.306	2.029	0.042	0.098	Glomeromycetes, Glomerales, Glomeraceae, Dominikia, Dominikia iranica
**OTU56**	1.438	0.656	0.297	2.210	0.027	0.073	Glomeromycetes, Glomerales, Glomeraceae, Septoglomus, Septoglomus constrictum
**OTU96**	1.659	0.703	0.307	2.289	0.022	0.066	Sordariomycetes
**OTU171**	1.447	0.800	0.305	2.623	0.009	0.032	NA
**OTU47**	2.314	1.07	0.334	3.206	0.001	0.007	Glomeromycetes, Glomerales, Claroideoglomeraceae
**OTU84**	1.738	1.188	0.322	3.694	0	0.001	Glomeromycetes, Glomerales, Glomeraceae, Rhizoglomus, Rhizoglomus irregulare SAF22
**OTU74**	1.829	1.291	0.333	3.877	0	0.001	Glomeromycetes, Glomerales, Glomeraceae, Rhizoglomus, Rhizoglomus irregulare SAF22
**OTU4**	84.543	1.480	0.530	2.792	0.005	0.020	Glomeromycetes, Glomerales, Glomeraceae, Rhizoglomus, Rhizoglomus irregulare SAF22
**OTU16**	15.497	4.579	0.389	11.771	0	0	Glomeromycetes, Glomerales, Glomeraceae, Rhizoglomus, Rhizoglomus irregulare SAF22
**OTU9**	30.702	5.074	0.442	11.482	0	0	Glomeromycetes, Glomerales, Glomeraceae, Rhizoglomus, Rhizoglomus irregulare SAF22
**OTU2**	241.62	7.024	0.429	16.371	0	0	Glomeromycetes, Glomerales, Glomeraceae, Rhizoglomus, Rhizoglomus irregulare SAF22

Supplementary Table 16: Number of AMF OTUs (Phylum Glomeromycota) in the root samples. Table shows the number of OTUs with the taxonomic assignment "Glomeromycota" for the control and inoculated samples, as well as their difference (delta). A positive number represents an increase in AMF OTUs as response to inoculation with SAF22 (44% of the fields) and a negative number a decrease in AMF OTUs (42% of the fields). The mean change in biodiversity across all fields is 0.46.

Field	OTU richness CTL	OTU richness AMF	delta (AMF-CTL)
**F01**	14	9	-5
**F04**	8	13	5
**F05**	4	5	1
**F06**	8	7	-1
**F07**	9	4	-5
**F08**	11	4	-7
**F09**	7	5	-2
**F10**	10	5	-5
**F12**	10	10	0
**F13**	5	6	1
**F14**	1	1	0
**F16**	15	19	4
**F19**	5	3	-2
**F21**	11	11	0
**F22**	8	3	-5
**F23**	1	3	2
**F24**	2	2	0
**F25**	7	8	1
**F26**	1	1	0
**F27**	4	6	2
**F28**	2	7	5
**F29**	3	7	4
**F30**	3	3	0
**F31**	8	3	-5
**F32**	6	7	1
**F33**	2	7	5
**F34**	6	3	-3
**F35**	0	3	3
**F36**	1	1	0
**F37**	3	5	2
**F38**	8	2	-6
**F39**	2	5	3
**F40**	6	7	1
**F42**	3	0	-3
**F43**	1	6	5
**F44**	7	5	-2
**F45**	6	5	-1
**F46**	2	5	3
**F47**	6	3	-3
**F48**	20	11	-9
**F49**	18	15	-3

**F50**	8	11	3
**F51**	9	10	1
**F52**	22	13	-9
**F53**	23	18	-5
**F54**	18	27	9
**F55**	16	13	-3
**F56**	16	19	3
**F58**	20	14	-6
**F59**	23	26	3
