# Supplementary material for: Soil microbiome indicators can predict crop growth response to large-scale inoculation with arbuscular mycorrhizal fungi
Source: Nat Microbiol. 2023 Nov 29;8(12):2277–89. doi: 10.1038/s41564-023-01520-w (PMC10730404; doi:10.1038/s41564-023-01520-w)
Supplement: Supplementary file 2 — Reporting Summary [file 41564_2023_1520_MOESM2_ESM.pdf]

nature portfolio

Corresponding author(s): Marcel van der Heijden
Klaus Schlaeppi

Last updated by author(s): Oct 3, 2023

Reporting Summary

Nature Portfolio wishes to improve the reproducibility of the work that we publish. This form provides structure for consistency and transparency in reporting. For further information on Nature Portfolio policies, see our Editorial Policies and the Editorial Policy Checklist.

Statistics

For all statistical analyses, confirm that the following items are present in the figure legend, table legend, main text, or Methods section.

n/a	Confirmed	
☐	☒	The exact sample size (*n*) for each experimental group/condition, given as a discrete number and unit of measurement
☐	☒	A statement on whether measurements were taken from distinct samples or whether the same sample was measured repeatedly
☐	☒	The statistical test(s) used AND whether they are one- or two-sided<br>*Only common tests should be described solely by name; describe more complex techniques in the Methods section.*
☐	☒	A description of all covariates tested
☐	☒	A description of any assumptions or corrections, such as tests of normality and adjustment for multiple comparisons
☐	☒	A full description of the statistical parameters including central tendency (e.g. means) or other basic estimates (e.g. regression coefficient) AND variation (e.g. standard deviation) or associated estimates of uncertainty (e.g. confidence intervals)
☐	☒	For null hypothesis testing, the test statistic (e.g. *F*, *t*, *r*) with confidence intervals, effect sizes, degrees of freedom and *P* value noted<br>*Give P values as exact values whenever suitable.*
☒	☐	For Bayesian analysis, information on the choice of priors and Markov chain Monte Carlo settings
☒	☐	For hierarchical and complex designs, identification of the appropriate level for tests and full reporting of outcomes
☒	☐	Estimates of effect sizes (e.g. Cohen's *d*, Pearson's *r*), indicating how they were calculated

Our web collection on statistics for biologists contains articles on many of the points above.

Software and code

Policy information about availability of computer code

Data collection	No software was used.
Data analysis	The code used to analyze the data is available under https://github.com/PMI-Basel/Lutz_et_al_Predicting_crop_yield.<br><br>All statistical analyses described below were carried out in R v.4.0.3 and plots were created using the R packages ggplot2 v.3.3.5, graphics v.4.0.3 or ggpubr v.0.4.0. Other R packages used: stats (v.4.0.3), psych v.2.1.9, reshape2 (v.1.4.4), randomForest (v.4.6-14), MASS (v.7.3.54), glmulti (v.1.0.8)<br><br>Inkscape v.092 was used for finalisation of figures.

For manuscripts utilizing custom algorithms or software that are central to the research but not yet described in published literature, software must be made available to editors and reviewers. We strongly encourage code deposition in a community repository (e.g. GitHub). See the Nature Portfolio guidelines for submitting code & software for further information.

Data

Policy information about availability of data

All manuscripts must include a data availability statement. This statement should provide the following information, where applicable:

- Accession codes, unique identifiers, or web links for publicly available datasets
- A description of any restrictions on data availability
- For clinical datasets or third party data, please ensure that the statement adheres to our policy

The raw sequencing data are stored in the European Nucleotide Archive (http:// www.ebi.ac.uk/ena) under accession number PRJEB53587 (soil microbiome) and PRJEB56590 (root microbiome). All other data are available in the supplementary material.

Human research participants

Policy information about studies involving human research participants and Sex and Gender in Research.

Reporting on sex and gender	NA
Population characteristics	NA
Recruitment	NA
Ethics oversight	NA

Note that full information on the approval of the study protocol must also be provided in the manuscript.

Field-specific reporting

Please select the one below that is the best fit for your research. If you are not sure, read the appropriate sections before making your selection.

☐ Life sciences ☐ Behavioural & social sciences ☒ Ecological, evolutionary & environmental sciences

For a reference copy of the document with all sections, see nature.com/documents/nr-reporting-summary-flat.pdf

Ecological, evolutionary & environmental sciences study design

All studies must disclose on these points even when the disclosure is negative.

Study description	We conducted inoculation experiments with arbuscular mycorrhizal fungi (AMF) in 54 arable fields and investigated the effect of AMF inoculation on maize yield. In order to assess the factors explaining inoculation success, a total of 52 physico-chemical soil parameters was analysed in combination with PacBio sequencing of the soil and root microbiome.
Research sample	Bulk soil at the beginning of the growth season and maize shoot and root samples at time of harvest
Sampling strategy	The field inoculations were carried out in three consecutive years in a total of 54 maize fields in northern Switzerland.
Data collection	Soil sampling took place before fertilisation of the fields and was performed using a half-cylindrical gouge auger (Eijkelkamp; effective auger body: 100 cm, Ø: 3 cm). Twenty soil cores were mixed to form composite samples and kept cold during transportation back to the laboratory.<br>At time of harvest, after approximately four to five months of plant growth, two plants from the centre of each of the eight plots per treatment and field (i.e. 16 plants in total) were cut 10 cm above the soil surface and their fresh weight was determined. Roots were collected and thoroughly washed with water, cut into pieces of approximately 1-2 cm length and well mixed.
Timing and spatial scale	Time of sampling: In 2018, 22 fields were inoculated between 23rd of April and 16th of May. In 2019, 25 fields were sampled between 18th of April and 7th of June. In 2020, 12 fields were sampled between 22nd of April and 16th of May.<br>Spatial scale: Northern Switzerland
Data exclusions	NA
Reproducibility	The experimental set-up was repeated in three consecutive years (however in different fields due to crop rotation).
Randomization	In 2018, a split plot design was used for practicality reasons. Each experimental field comprised 12 maize rows à 24 m, with the spacing between two maize rows being 75 cm. Fertiliser types (NK and NPK; N=nitrogen, P=phosphorus, K=potassium) were randomly assigned to whole plots and inoculum types (control and AMF) were randomly assigned to split plots within each whole

	plot. In 2019 and 2020 a randomized complete block design was used with 8 blocks. It was ensured that there were at least three maize rows serving as a buffer zone between the first inoculated maize row and the edge of the field, to avoid edge effects.
Blinding	Sample codes were used during experiments and analyses that did not reveal their origin.

Did the study involve field work? ☒ Yes ☐ No

Field work, collection and transport

Field conditions	Weather conditions varied slightly across the three years and 54 fields. However, in order to reduce variation, samples were collected during the same growth stage across fields and years.
Location	Northern Switzerland
Access & import/export	Inoculation trials and sampling took place in agreement with farmers.
Disturbance	Disturbance caused by soil sampling was minimal.

Reporting for specific materials, systems and methods

We require information from authors about some types of materials, experimental systems and methods used in many studies. Here, indicate whether each material, system or method listed is relevant to your study. If you are not sure if a list item applies to your research, read the appropriate section before selecting a response.

Materials & experimental systems

n/a	Involved in the study	
☒	☐	Antibodies
☒	☐	Eukaryotic cell lines
☒	☐	Palaeontology and archaeology
☒	☐	Animals and other organisms
☒	☐	Clinical data
☒	☐	Dual use research of concern

Methods

n/a	Involved in the study	
☒	☐	ChIP-seq
☒	☐	Flow cytometry
☒	☐	MRI-based neuroimaging
